# Therapeutic avenues in bone repair: Harnessing an anabolic osteopeptide, PEPITEM, to boost bone growth and prevent bone loss

**DOI:** 10.1016/j.xcrm.2024.101574

**Published:** 2024-05-21

**Authors:** Jonathan W. Lewis, Kathryn Frost, Georgiana Neag, Mussarat Wahid, Melissa Finlay, Ellie H. Northall, Oladimeji Abudu, Samuel Kemble, Edward T. Davis, Emily Powell, Charlotte Palmer, Jinsen Lu, G. Ed Rainger, Asif J. Iqbal, Myriam Chimen, Ansar Mahmood, Simon W. Jones, James R. Edwards, Amy J. Naylor, Helen M. McGettrick

**Affiliations:** 1 Institute of Inflammation and Ageing, University of Birmingham, Birmingham B15 2WB, UK; 2 Institute of Cardiovascular Sciences, University of Birmingham, Birmingham B15 2TT, UK; 3 Royal Orthopaedic Hospital, Bristol Road, Birmingham B31 2AP, UK; 4 Botnar Research Centre, University of Oxford, Oxford OX3 7LD, UK; 5 Department of Trauma and Orthopaedics, University Hospitals NHS Foundation Trust, Edgbaston, Birmingham B15 2GW, UK

**Keywords:** osteoblast, osteoclast, bone mineral density, bone, osteoporosis, PEPITEM, NCAM-1, rheumatoid arthritis, b-catenin, OPG

## Abstract

The existing suite of therapies for bone diseases largely act to prevent further bone loss but fail to stimulate healthy bone formation and repair. We describe an endogenous osteopeptide (PEPITEM) with anabolic osteogenic activity, regulating bone remodeling in health and disease. PEPITEM acts directly on osteoblasts through NCAM-1 signaling to promote their maturation and formation of new bone, leading to enhanced trabecular bone growth and strength. Simultaneously, PEPITEM stimulates an inhibitory paracrine loop: promoting osteoblast release of the decoy receptor osteoprotegerin, which sequesters RANKL, thereby limiting osteoclast activity and bone resorption. In disease models, PEPITEM therapy halts osteoporosis-induced bone loss and arthritis-induced bone damage in mice and stimulates new bone formation in osteoblasts derived from patient samples. Thus, PEPITEM offers an alternative therapeutic option in the management of diseases with excessive bone loss, promoting an endogenous anabolic pathway to induce bone remodeling and redress the imbalance in bone turnover.

## Highlights

• PEPITEM exerts anabolic osteogenic activity to regulate osteoblast-osteoclast coupling
• PEPITEM acts directly on osteoblasts to promote formation of new and stronger bone
• PEPITEM stimulates an inhibitory paracrine loop via OPG to limit bone resorption
• PEPITEM therapy halts disease-induced bone loss *in vivo*

## Graphical abstract

**Figure:**
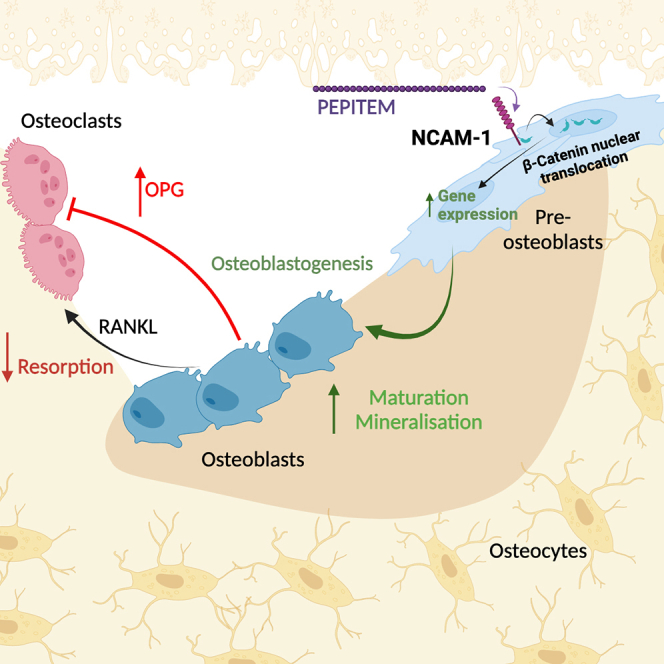

## Teaser

Lewis and Frost et al. identify the anabolic activity of an endogenous osteopeptide (PEPITEM), revealing the cellular and molecular mechanisms by which PEPITEM regulates bone remodeling *in vitro* and in preclinical disease models, to promote new bone formation. They suggest that PEPITEM offers an alternative therapeutic option for bone loss diseases.

## Introduction

Bone is a highly active organ, undergoing continuous osteoblast-induced bone formation and osteoclast-mediated bone resorption throughout life. The process of bone remodeling is orchestrated by cross-talk among osteoblasts, osteoclasts, and osteocytes acting in concert to maintain structural integrity, repair damage, and respond to changes in activity and load.^1^ Dysregulation of these pathways underpins numerous musculoskeletal (MSK) diseases where excessive bone resorption (e.g., osteoporosis, rheumatoid arthritis, periodontal disease, cancer-bone metastases) or abnormal bone formation (e.g., ankylosing spondylitis; heterotopic ossification) results in permanent loss of function, pain, increased risk of fracture, and frailty.^2^ Osteoporosis is the most common bone disease globally, affecting over 54 million individuals in the United States and accounting for 3 million broken bones at a cost of ∼$26 billion per annum.^3^ There are no cures for bone damage. Existing therapies have predominantly focused on slowing the rate of bone damage (e.g., bisphosphonates; denosumab, saracatinib), with only a handful of drugs able to promote bone repair currently approved (e.g., parathyroid hormone—PTH or romosozumab).^4^^,^^5^ Due to poor patient response, poor drug compliance, and drug-induced microfractures leading to atypical femur fractures, there is an urgent need to develop a new suite of therapies that lead to bone repair and regeneration in patients with MSK diseases to restore tissue homeostasis and functional integrity.

Approximately 10% of all bone in the human body is replaced annually, through a series of tightly coordinated sequential steps (reviewed by Kenkre and Bassett^6^ and Delaisse et al.^7^). Hematopoietic myeloid cells (monocytes) recruited to the bone differentiate into mononuclear osteoclast precursors in response to M-CSF (macrophage colony stimulating factor) and later RANKL (receptor activator of nuclear factor-κB ligand) stimulation, before fusing together to form multinucleated mature osteoclasts. Within osteoclast resorption pits, locally released chloride and hydrogen ions dissolve the bone mineral, while proteases (matrix metalloproteinase [MMP], cathepsin K) digest the collagen matrix resulting in bone resorption. Replacement of this resorbed bone is performed by osteoblasts, derived from mesenchymal stem cells (MSCs), that become committed to the osteoblast lineage upon activation of the transcriptional regulator Runx2. As osteoblast precursors start to differentiate, they express increased amounts of collagen (*COL1A1*) and matrix proteins, which are deposited as an unmineralized osteoid. Maturing osteoblasts subsequently release alkaline phosphatase (ALP) into the collagen-rich matrix resulting in the deposition of hydroxyapatite crystals, mineralization of the matrix, and formation of new bone.

Physiological bone remodeling is tightly regulated by the intricate cross-talk between osteoblasts and osteoclasts at the bone surface and via signaling from osteocytes embedded within the bone tissue itself. Osteoblasts and osteocytes release both positive (RANKL, M-CSF) and negative (osteoprotegerin [OPG]) regulators of osteoclastogenesis to control the rate of bone resorption.^8^ Conversely, osteoclasts and osteocytes produce anabolic stimuli that include sphingosine 1-phosphate,^9^ Wnt/β-catenin proteins, and bone morphogenic proteins^10^ that induce osteoblast precursor recruitment, differentiation, and survival. The highly conserved and ubiquitously expressed seven isoforms of the human 14-3-3 family act as adaptor proteins influencing the function of other proteins, and thus a multitude of signaling pathways, to regulate cellular responses (reviewed by Obsilova and Obsil^11^). The name 14-3-3 derives from the elution fraction (14^th^) and subsequent position (3.3) on starch electrophoresis gel when the family was first identified in brain tissue.^12^ Of particular relevance to bone homeostasis, two 14-3-3 family members have been reported to differentially regulate osteoblast function, with 14-3-3β^13^ significantly reducing and 14-3-3ξ enhancing osteoblastogenesis.^14^ We have previously identified a bioactive 14-amino acid peptide (PEPITEM) cleaved from 14-3-3ξ^15^, which regulates the migration of monocytes (osteoclast precursors) into non-bone tissues during inflammation.^16^^,^^17^ Here we investigated the ability of PEPITEM to directly influence bone remodeling under homeostatic conditions and subsequently the therapeutic efficacy of PEPITEM in models of excessive bone loss.

## Results

### PEPITEM enhances bone formation and strength under homeostatic conditions

**Figure 1:**
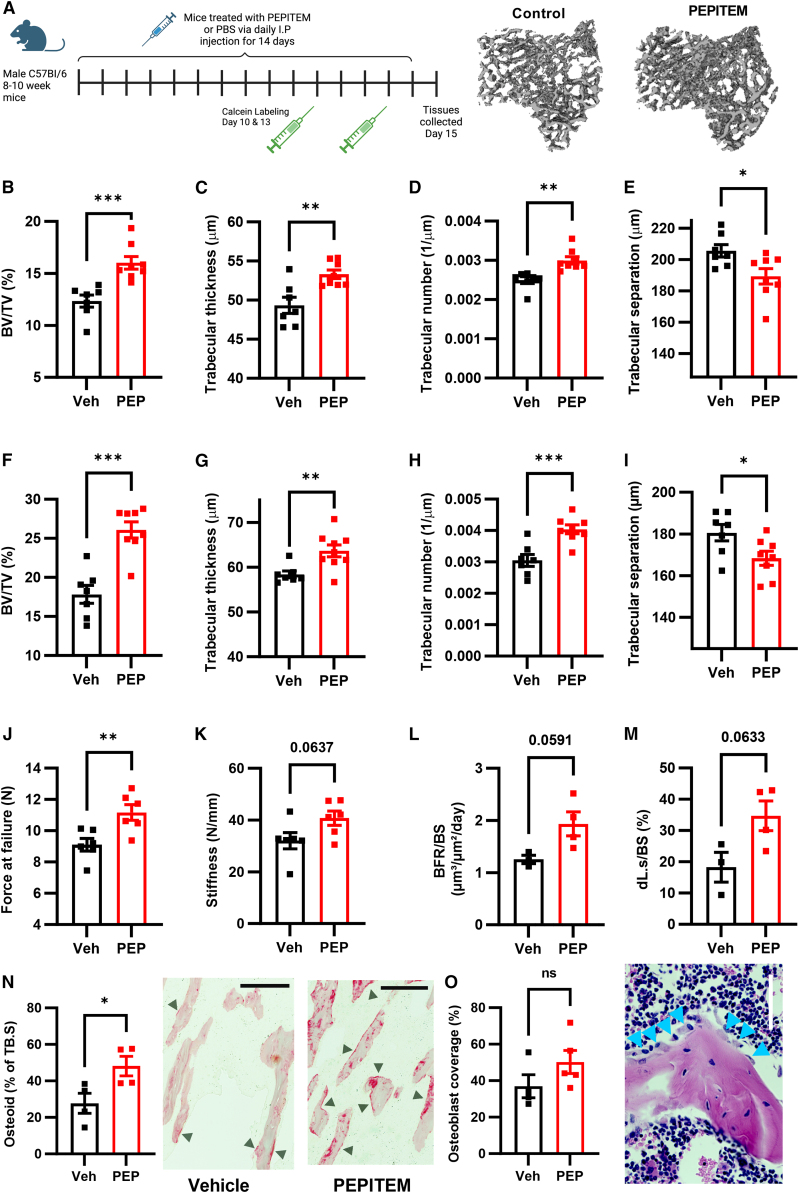
PEPITEM enhances bone formation and strength under homeostatic conditions Healthy young mice injected with vehicle control (Veh, black) or PEPITEM-PEG (PEP, red) and then (B–E, L and M) tibiae, (F–I) vertebrae (L4–6), or (J and K) femurs were analyzed. (A) Schematic representation of experiment and representative microCT images of tibiae trabecular bone. (B and F) Percentage trabecular bone volume (BV/TV). *n* = 7–8 mice from three independent experiments. (C and G) Trabecular thickness in μm. *n* = 7–8 mice from three independent experiments. (D and H) Trabecular number per μm. *n* = 7–8 mice from three independent experiments. (E and I) Trabecular separation in μm. *n* = 7–8 mice from three independent experiments. (J and K) Femurs were subject to 3-point bend to assess (J) force at failure in N/mm and (K) stiffness in N for two femurs per mouse, *n* = 3 mice from one independent experiment. (L and M) Dynamic histomorphometry of (L) bone formation rate normalized to bone surface perimeter (BFR/BS) as μm^3^/μm^2^/day and (M) length of double calcein labels normalized to total bone surface perimeter (dL.s/BS) as a percentage. *n* = 3–4 for two independent experiments. (N) Percentage of trabecular bone surface stained with picrosirius red representing osteoid compared with total trabecular bone surface area. *n* = 4 mice from two independent experiments (OB/BS). (O) Number of osteoblasts in the tibiae from mice treated expressed as surface area of bone covered by osteoblasts as percentage of the total bone surface area (Ob.S/BS). *n* = 4–5 mice per group from two independent experiments. Representative image, where cyan arrows point to osteoblasts. Scale bar, 50 μm. Data are mean ± SEM. ∗*p* < 0.05, ∗∗*p* < 0.01, and ∗∗∗*p* < 0.001 by unpaired t test.

Initially we examined whether PEPITEM intrinsically regulated bone remodeling under basal conditions over a 2-week period (Figure 1A). PEPITEM therapy significantly increased bone volume (BV/TV), trabecular number, and thickness in both the tibia (Figures 1B–1D) and vertebrae (Figures 1F–1H) of adult mice, indicating that PEPITEM promotes bone formation. As expected, we observed a concomitant decrease in the gaps between individual trabeculae (trabecular separation [Figures 1E and I]), as the increased trabeculae created a denser, more interconnected trabecular network. Cortical bone turnover takes much longer than trabecular bone: ∼4–6 weeks.^18^ As expected, we observed no changes in the cortical bone following 2 weeks of PEPITEM treatment, but we did observe a significant increase in cortical bone parameters following 4 weeks of treatment (Figure S1). When comparing these findings with existing drugs targeting the bone, the effect size for PEPITEM on BV/TV at 2 weeks is comparable to that seen following treatment with the bisphosphonate zoledronic acid for 3 weeks^19^ or PTH for up to 4 weeks.^20^ Thus indicating PEPITEM is as efficient at inducing bone formation compared with current standard of care.

The enhanced trabecular parameters induced by PEPITEM translated to increased bone strength, with PEPITEM therapy significantly increasing the force at which bones fracture (Figure 1J) and tends to enhance bone stiffness (Figure 1K) compared with control-treated animals. Dynamic histomorphometry revealed increases in both the rate of bone formation (Figure 1L) and the overall mineralized bone surface (Figure 1M), demonstrating that PEPITEM induces bone mineralization leading to increased bone strength. Further supporting this, PEPITEM significantly increased the deposition of osteoid when compared with control animals (Figure 1O). Furthermore, PEPITEM treatment tended to increase osteoblast numbers when compared with vehicle, albeit not significantly (Figure 1P), suggesting increased bone mineralization was predominantly a result of increased osteoblast activity rather than absolute number. No detectable effect on the levels of the bone formation serum markers—soluble procollagen type 1 N-terminal propeptide (P1NP)—was observed in PEPITEM-treated compared with control-treated mice in this experiment (Figure S2). Collectively these data reveal the pro-osteogenic actions of PEPITEM in the absence of inflammation.

### PEPITEM acts directly on osteoblasts to enhance bone mineralization and limit bone resorption

**Figure 2:**
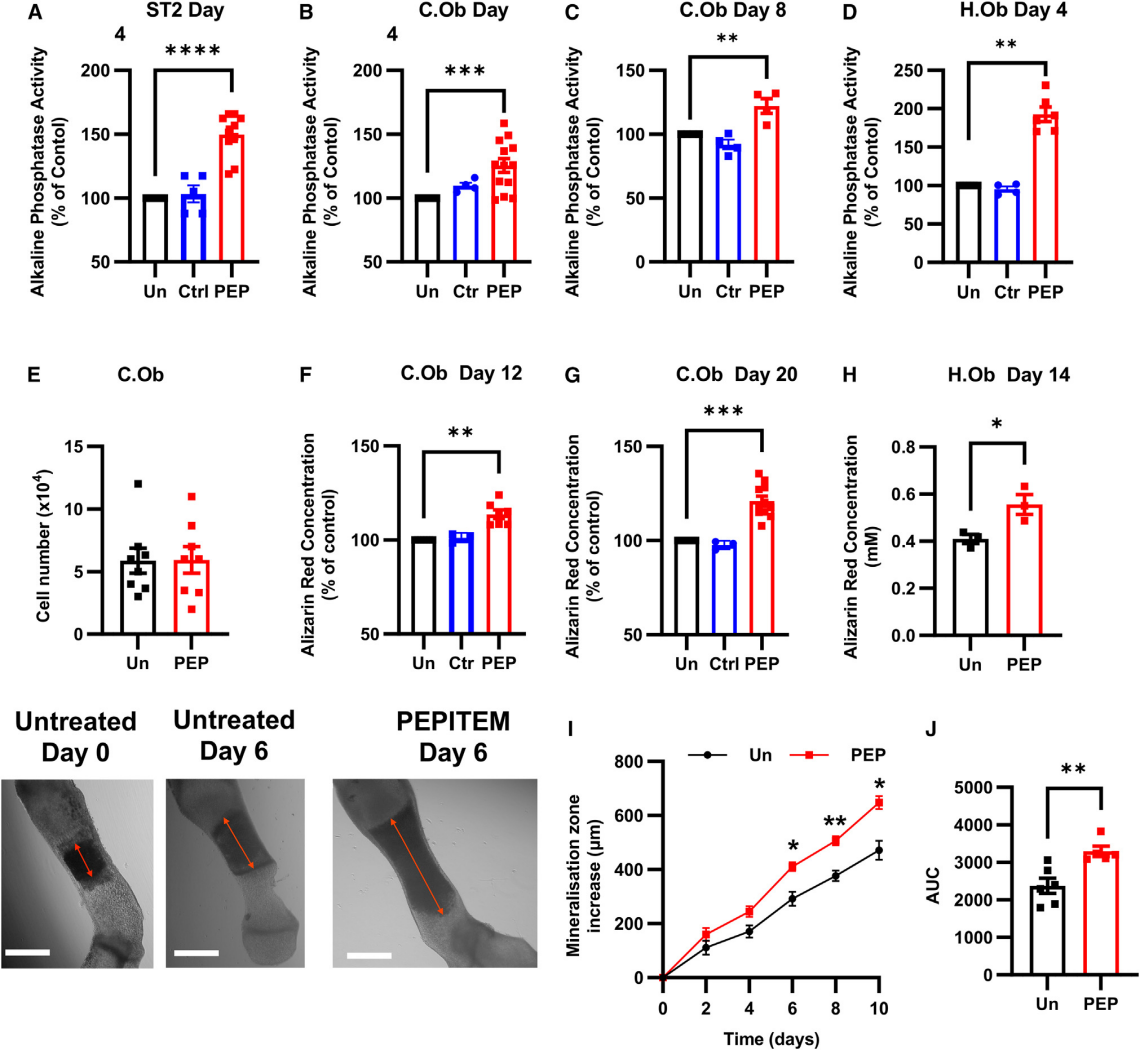
PEPITEM acts directly on osteoblasts to enhance bone mineralization and limit bone resorption (A–D) Osteoblasts were cultured in osteogenic media (untreated, Un, black) supplemented with a control peptide (Ctr, blue) or PEPITEM (PEP, red). Alkaline phosphatase activity for (A) ST2 cells (*n* = 5–10), (B and C) primary calvarial (*n* = 4–13), or (D) hFOb 1.19 cells (*n* = 4–6) measured at day (A, B, and D) 4 or (C) 8 and normalized to percentage of untreated control. In (A–C), Kruskal-Wallis shows a significant effect of treatment, *p* < 0.01. (E–H) (E) Proliferation of primary calvarial osteoblasts expressed as cell count × 10^4^ cells, *n* = 8. Alizarin red concentration extracted from (F and G) calvarial osteoblasts or (H) hFOb 1.19 cells at day (F) 12 (*n* = 3–7) (G) 20 (*n* = 3–11), or (H) 14 (*n* = 3) expressed as percentage of untreated control or mM. In (F) and (G), Kruskal-Wallis, shows a significant effect of treatment, *p* < 0.01. (I and J) Mineralization of metatarsal bones cultured without (untreated, un, black) or with PEPITEM (PEP, red) as representative images at different time points or plotted as (I) increase in mineral zone in μm or (J) area under the curve (AUC), *n* = 5–6. In (I), ANOVA shows a significant effect of treatment, *p* < 0.001. Data are mean ± SEM. ∗*p* < 0.05, ∗∗*p* < 0.01, ∗∗∗*p* < 0.001, and ∗∗∗∗*p* < 0.0001 by (A–C, F, and G) Dunn post-test, (D and H) Wilcoxon, (I) Bonferroni post-test between treatments for each time point, or (J) paired t test.

Osteoblasts and osteoclasts work in concert to balance bone formation and resorption—thus an increase in trabecular bone could result from an increase in osteoblast activity or a decrease in osteoclast activity. Using alkaline phosphatase activity as an early marker of osteoblast maturation and alizarin red to visualize the later occurrence of bone mineralization, we next investigated the direct effect of PEPITEM on osteoblast function *in vitro*. PEPITEM significantly increased alkaline phosphatase activity in the murine ST2 osteoblast cell line (Figure 2A), primary murine calvarial osteoblasts isolated from male and female mice (Figures 2B and 2C) and the human hFOb 1.19 osteoblast cell line (Figure 2D) in culture. Of note, PEPITEM had no effect on cell number when compared with untreated controls (Figure 2E), indicating the changes in ALP are not due to more cells being present. This heightened PEPITEM-induced ALP expression resulted in significantly enhanced osteoblast mineral production over time, as detected by increases in alizarin red concentration (Figures 2F–2H). No changes were seen in osteoblast maturation or mineralization when cells were treated with an alternative 14aa peptide sequence within 14-3-3ξ (parent protein of PEPITEM, Figures 2A–2D, 2F, and 2G), indicating that it is PEPITEM itself that exhibits osteogenic properties. Supporting these *in vitro* findings, PEPITEM significantly enhanced mineral formation within intact metatarsal bones cultured *ex vivo* (Figures 2I and 2J). These findings demonstrate that PEPITEM acts directly on osteoblasts in the bone microenvironment to increase mineral deposition and together with the increases in bone formation rate and osteoid deposition observed *in vivo* collectively support the anabolic role of PEPITEM in bone formation. Several questions remain unanswered: what is the receptor for PEPITEM on osteoblasts and can PEPITEM influence osteoclast function?

### PEPITEM interaction with NCAM-1 leads to β-catenin translocation in osteoblasts

In the inflamed blood vascular bed, we have previously demonstrated that PEPITEM interacts with the adhesion molecule cadherin-15 (CDH15) on blood vascular endothelial cells to mediate its downstream immunomodulatory effects via sphingosine-1-phosphate (S1P).^15^ While primary murine and human osteoblasts express CDH15 transcript and protein (Figure S3A–S3C), treating primary murine osteoblasts with a CDH15 agonistic antibody had no effect on bone mineralization (Figure S3D), suggesting that CDH15 is not responsible for mediating the actions of PEPITEM on osteoblasts. Moreover, increasing concentrations of S1P caused a dose-dependent reduction in alkaline phosphate activity, and thus osteoblast activity (Figure S3E), in contrast to the increase observed with PEPITEM. Collectively, these data strongly indicate that the osteogenic actions of PEPITEM are triggered by a previously undescribed mechanism, which is distinct from the immunopeptide actions described for leukocyte trafficking during an inflammatory challenge.

**Figure 3:**
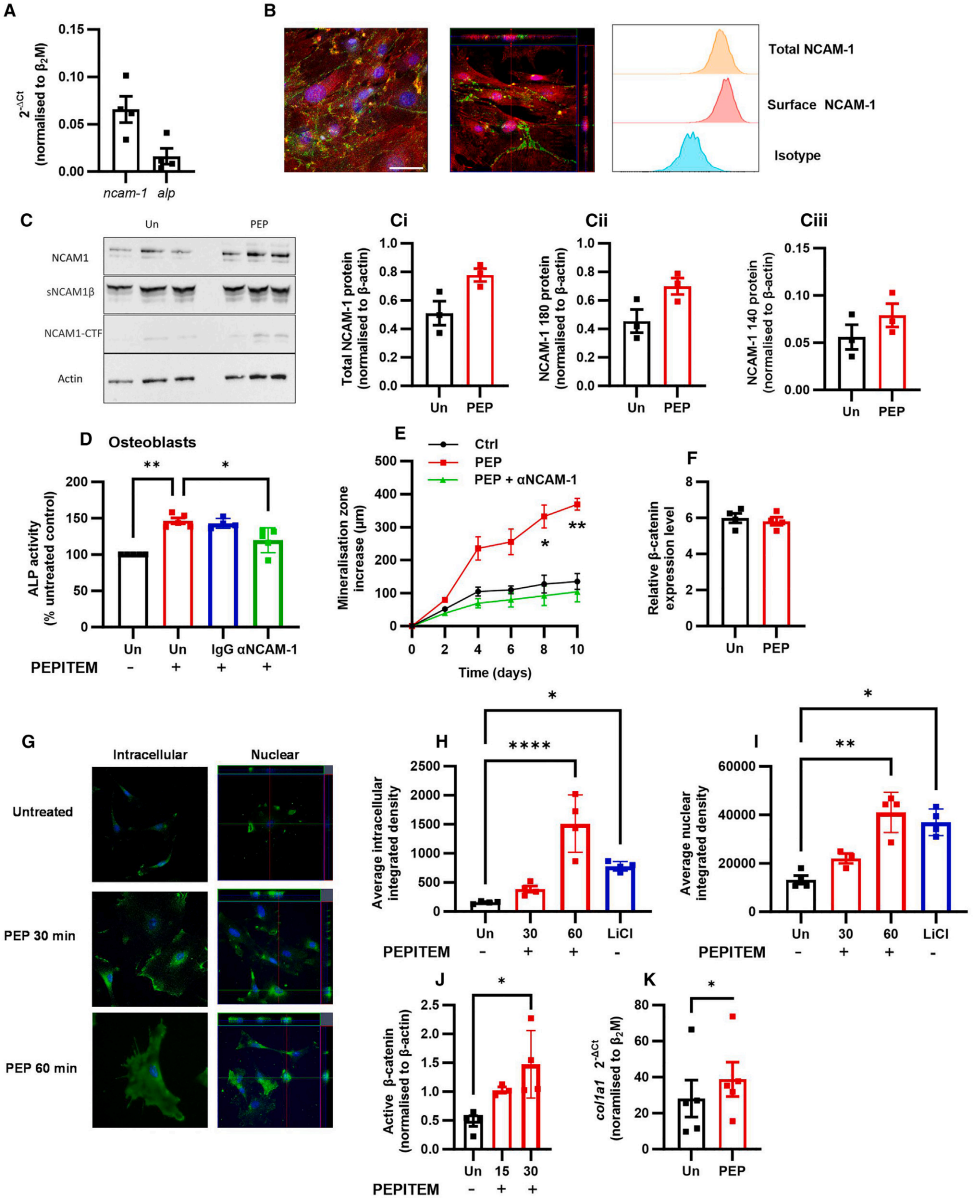
PEPITEM signaling through NCAM-1 on osteoblasts leads to b-catenin translocation (A, F, and K) Gene expression of (A) *ncam-1, alpl,* (F) *β-catenin*, and (K) *col1a1* in osteoblasts expressed as 2^−ΔCT^ of β_2_M or relative expression level from bulk-RNA-sequencing, *n* = 3–6. (B) Representative confocal images of NCAM-1 (green), RUNX2 (red) expression, and DAPI (blue) from two independent experiments and histograms of total and surface NCAM-1 expression as assessed by flow cytometry. (C) Representative gel for 3 independent experiments analyzing (Ci) total, (Cii) 180, or (Ciii) 140 NCAM-1 isoforms following treatment without (Un) or with PEPITEM (PEP) normalized to β-actin, *n* = 3. (D and E) Osteoblasts or metatarsals were left untreated (Un, black) or treated with PEPITEM (PEP, red), IgG control antibody (blue), or anti-NCAM-1 antibody (green). (D) Alkaline phosphatase activity normalized to percentage of untreated osteoblasts, *n* = 4. (E) Metatarsal mineralization increased in μm over time, *n* = 3. (G–J) Osteoblasts were untreated (Un, black) or treated with PEPITEM (PEP, red) or lithium chloride (LiCl, blue). (G) Representative images of β-catenin intracellular and nuclear expression pattern (green). Average β-catenin (H) intracellular or (I) nuclear localisation at 30 and 60 min expressed as integrated density, *n* = 4–5. (J) Active β-catenin protein expression at 15 and 30 min as band intensity (AUC) relative to β-actin loading control, *n* = 3–4. (D) Kruskal-Wallis or (E, G, and H) ANOVA shows a significant effect of treatment, *p* < 0.001. Data are mean ± SEM. ∗*p* < 0.05, ∗∗*p* < 0.01, and ∗∗∗∗*p* < 0.0001 by (D) Dunn or (E, G, and H) Bonferroni post-test, or (J) paired t test. Scale bar, (B) 50 μm; (G) 100 μm.

To identify a PEPITEM receptor on osteoblasts, we used a biotin-conjugated PEPITEM to “fish” for potential binding partners on the cell surface of calvarial osteoblasts. This peptide showed pro-osteogenic activity, inducing osteoblast maturation and mineralization to a similar amount seen by native PEPITEM (Figure S4), indicating that biotinylation did not inhibit PEPITEM’s activity. PEPITEM-bound proteins eluted from osteoblast lysates were analyzed by mass spectrometry. Comparative analysis allowed exclusion of molecules identified in both control peptide and PEPITEM samples, and data were further filtered to include only those proteins associated with a membrane expression pattern using isoelectric point (Table S1). Of the top 10 hits, only two are known membrane proteins: NCAM-1 (CD56)^21^ and EHD2 (EH domain containing protein 2).^22^ Of these only NCAM-1 has previously been shown to be involved in osteoblastogenesis,^21^ although expression was transient and lost as MSC differentiate into osteoblasts.^23^^,^^24^ We detected both gene (Figure 3A) and protein expression of NCAM-1 in primary calvarial osteoblasts (Figures 3B and 3C), with western blot analysis revealing the presence of different NCAM-1 isoforms in osteoblasts—all of which increased in expression following PEPITEM treatment for 8 days (Figure 3C). To determine whether PEPITEM-enhanced osteoblast activity required NCAM-1, we treated osteoblasts with an NCAM-1 function blocking antibody or relevant immunoglobulin (Ig)G control in the presence of PEPITEM and assessed alkaline phosphatase activity. Inhibiting NCAM-1 blocked the effect of PEPITEM (Figure 3D), suggesting a role of NCAM-1 in mediating the effects of PEPITEM. Similarly, anti-NCAM-1 blocked PEPITEM-induced mineralization within intact metatarsal bones cultured *ex vivo* when compared with IgG1 treated controls (Figure 3E).

Previous evidence has shown that NCAM-1 regulation of osteoblast differentiation was mediated through Wnt/β-catenin and PI3K-AKT signaling pathways.^21^ While PEPITEM had no effect on β-catenin gene expression (Figure 3F), we observed a significant increase in the amount of intracellular β-catenin protein in osteoblasts following a 60-min incubation with PEPITEM (Figures 3G and 3H). This change was ∼1.9-fold higher than that observed with a known inducer of the β-catenin signaling pathway, lithium chloride.^25^ In the presence of a Wnt ligand, β-catenin is known to translocate to the nucleus where it acts as a co-activator of the Wnt response elements in target genes.^26^^,^^27^ Similarly, PEPITEM induced β-catenin nuclear translocation within 60 min to a comparable level as seen with lithium chloride (Figures 3G and 3I), strongly suggesting this pathway mediates PEPITEM-enhanced osteoblast activity. Further supporting this, we observed an increase in β-catenin activation within 15 min of PEPITEM treatment, reaching significance by 30 min (Figure 3J). Bulk RNA-sequencing analysis revealed 75 differentially regulated genes (DEGs) up-regulated and 36 down-regulated in osteoblasts after 6 h of treatment with PEPITEM compared with cells treated with control peptide (Tables S2, and S3). Of note, 17 of the 75 up-regulated DEGs were associated with osteoblast differentiation and skeletal development, including *col1a1.* Indeed, *col1a1* was significantly increased in response to PEPITEM following 6 h of treatment (Figure 3K), thus confirming induction of gene expression in response to β-catenin activation and nuclear translocation.

**Figure 4:**
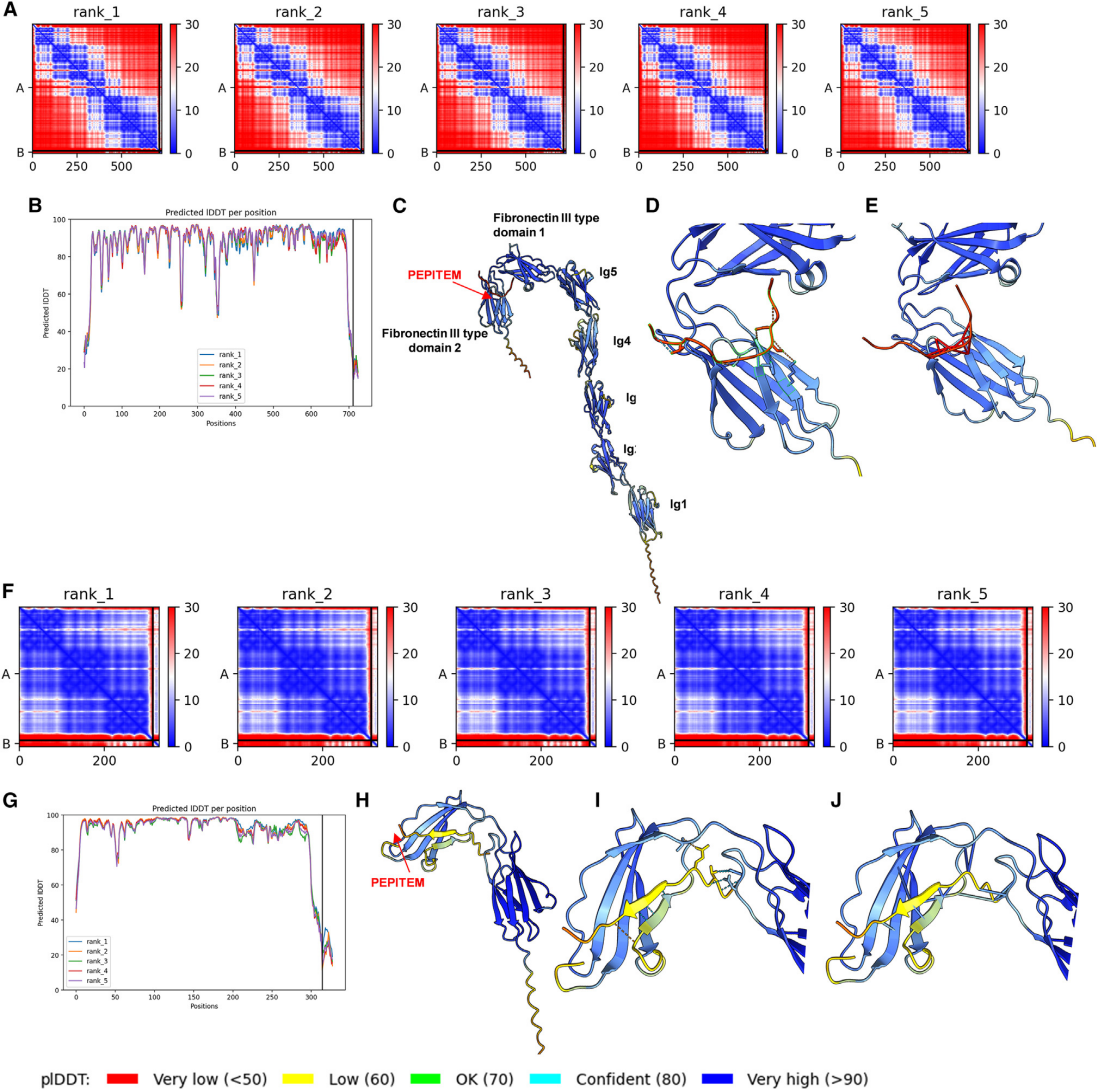
3-D predictive modeling of PEPITEM interaction with NCAM-1 (A–E) Full-length NCAM-1 extracellular domain or (F–J) fibronectin-III like domain were run with PEPITEM in (A and B, F and G) AlphaFold-Multimer followed by analyzed of rank 1 model (C–E and H–J) using ChimeraX. Predicted aligned error heatmap for five models of (A) NCAM-1 or (F) fibronectin-III like domain and PEPITEM, where blue and red indicate low or high error, respectively. (B and G) pLDDT score for each residue in the predicted models for (B) NCAM-1 or (G) fibronectin-III like domain. (C and H) ChimeraX modeling of rank 1 model for PEPITEM interactions with (C) NCAM-1 or (H) fibronectin-III colored by pLDDT. (D and E, I and J) Magnified view of binding location of PEPITEM on NCAM-1 revealing (D) four and (I) nine predicted hydrogen bonds—high likelihood (blue), low (orange)—and (E) 17 and (J) 13 pseudobonds based on a distance of 3 Å, confidence indicated by pLDDT color as above.

To further ascertain whether NCAM-1 is a receptor for PEPITEM, we used the AlphaFold2_Multimer neural network^28^ coupled with ChimeraX^29^ to generate five predicted 3-D models of PEPITEM binding/interacting with NCAM-1 (Figures 4A and 4B). NCAM-1 structurally has five IgG-like domains and two fibronectin-III-like domains.^30^ Predicted model five indicates PEPITEM binding to the first fibronectin domain within the extracellular portion of NCAM-1 (Figures 4C–4E, and Table S4). We also created 3-D models of PEPITEM interacting a peptide fragment of NCAM-1 containing the two fibronectin-III-like domains and IgG-like domain 5, observing much stronger predicted residue interactions compared with those obtained with the full NCAM-1 protein (Figures 4F and 4G). In this model, PEPITEM is interacting with the second fibronectin-III-like domain, with higher alignment scores (pLDDT score >70) than seen when PEPITEM is modeled with full-length NCAM-1 (Figures 4H–4J, and Table S4). Given EHD2 was also identified as a potential PEPITEM-binding partner expressed on the surface of osteoblasts, we created 3-D models predicting PEPITEM-End2 interactions (Figure S5). EHD2 consists of an N-terminal extended GTPase domain, a helical domain, and a C-terminal Eps15-homology domain.^31^ While *in silico* analysis suggests PEPITEM binds to the GTPase domain of EHD2, the predicted scores (pLDDT score 70 > 50) were lower than those seen in the NCAM-1-PEPITEM models (Figure S5). The combination of the predictive 3-D models and functional studies strongly suggest that PEPITEM mediates its effects via the NCAM-1-β-catenin signaling pathway, while also revealing a second potential binding partner in EHD2.

### PEPITEM acts indirectly on osteoclasts to reduce bone resorption

**Figure 5:**
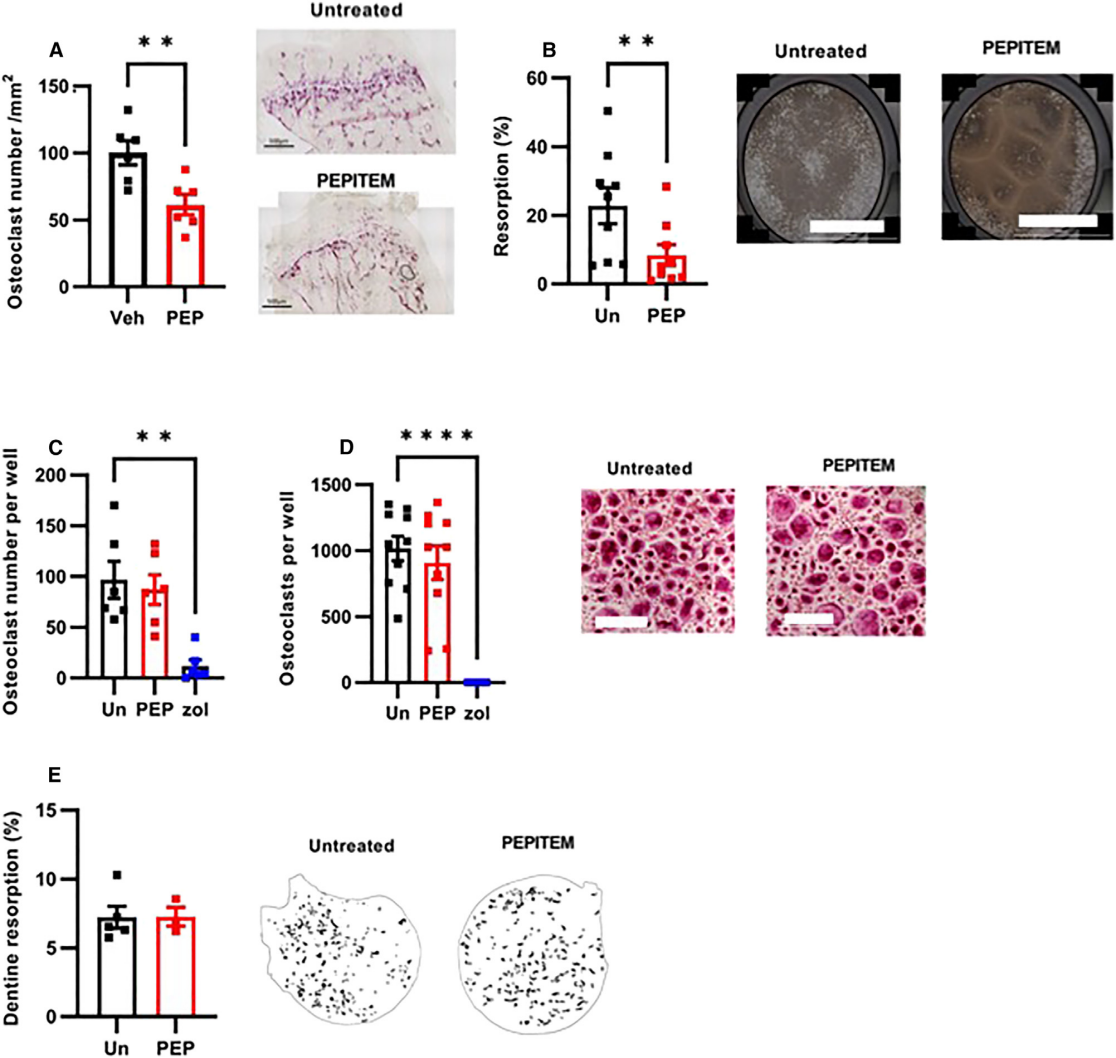
PEPITEM acts indirectly on osteoclasts to reduce bone resorption (A) Number of TRAP-positive multinucleated cells in the tibiae from mice treated with control (Veh, black, *n* = 6) or PEPITEM-PEG (PEP, red, *n* = 6) expressed as number of osteoclasts per mm^2^, ∗∗*p* < 0.05 by unpaired t test. (B) Osteoclast resorption on hydroxyapatite plates as percentage of total area for whole murine bone marrow cells left untreated (Un, black, *n* = 9) or with PEPITEM (PEP, red, *n* = 9). ∗∗*p* < 0.05 by paired t test. (C and D) Number of TRAP-positive osteoclasts cells per well differentiated from (C) RAW264.7 (*n* = 6) or human peripheral blood monocytes (*n* = 10) either left untreated (Un, black) or treated with PEPITEM (PEP, red) or with zoledronic acid (zol, blue). ANOVA showed a significant effect of treatment on osteoclast number, *p* < 0.01. ∗∗*p* < 0.01 and ∗∗∗∗*p* < 0.0001 by Dunnett post-test. (E) Human peripheral blood monocytes derived osteoclast resorption of dentine slices calculated from image masks (see inserts) as percentage of total area following treatment without (untreated, Un, black, *n* = 6) or with PEPITEM (PEP, red, *n* = 3). Data are mean ± SEM. Scale bar, (A and D) 500 μm; (B) 1,000 μm.

The interplay between osteoblast and osteoclast activity is carefully controlled, where increases in osteoblast activity are often matched with enhanced osteoclastogenesis (leading to increased bone resorption) to ensure balance is maintained. For this reason, it was necessary to assess the impact of PEPITEM on osteoclast function. Analysis of bone sections from mice treated with PEPITEM revealed a significant reduction in osteoclast numbers when compared with the treatment controls (Figure 5A), as measured by the presence of tartrate-resistant acid phosphatase (TRAP)-positive multinucleated cells. Similarly, PEPITEM significantly reduced mineral resorption when whole murine bone marrow was cultured with osteoclast-stimulating factors (M-CSF and RANKL) on specialist hydroxyapatite-coated plates (Figure 5B). As the action of PEPITEM appeared to also influence osteoclast number and function, we subsequently assessed whether this was mediated by direct action on the osteoclasts or the consequence of paracrine signaling from a neighboring cell type (e.g., osteoblast) that would be present in the *in vivo* samples and in the whole bone marrow extract assay.

Osteoclasts are derived from peripheral blood CD14^+^ monocytes following stimulation with RANKL and M-CSF. If PEPITEM binds directly to pre-osteoclasts to influence osteoclastogenesis, we predicted a reduction in osteoclast formation and mineral resorption in its presence. However, we observed no difference in the number of TRAP-positive osteoclasts when either the murine macrophage cell line RAW264.7 (Figure 5C) or primary human monocytes (Figure 5D) were differentiated into osteoclasts in the presence of PEPITEM. Induction of osteoclastogenesis was confirmed by significant up-regulation of the osteoclast genes *ACP5, ATP6V1B1*, and *CTSK* in primary human monocytes treated with RANKL (Figure S6). By contrast, a known inhibitor of osteoclasts (the bisphosphonate, zoledronic acid) significantly reduced the number of murine and human-derived osteoclasts (Figures 5C and 5D). Furthermore, PEPITEM had no effect on osteoclast resorption capacity, with the same degree of dentine resorption seen when compared with untreated cells (Figure 5E). These data clearly show that PEPITEM has no direct effects on osteoclast differentiation or activity and indicates that paracrine signaling cascades induced by PEPITEM within the bone microenvironment are likely to be responsible for the reduction in osteoclast number observed *in vivo* and in the whole murine bone marrow culture experiments.

### PEPITEM induces osteoblasts to release a soluble mediator, which inhibits osteoclast function

**Figure 6:**
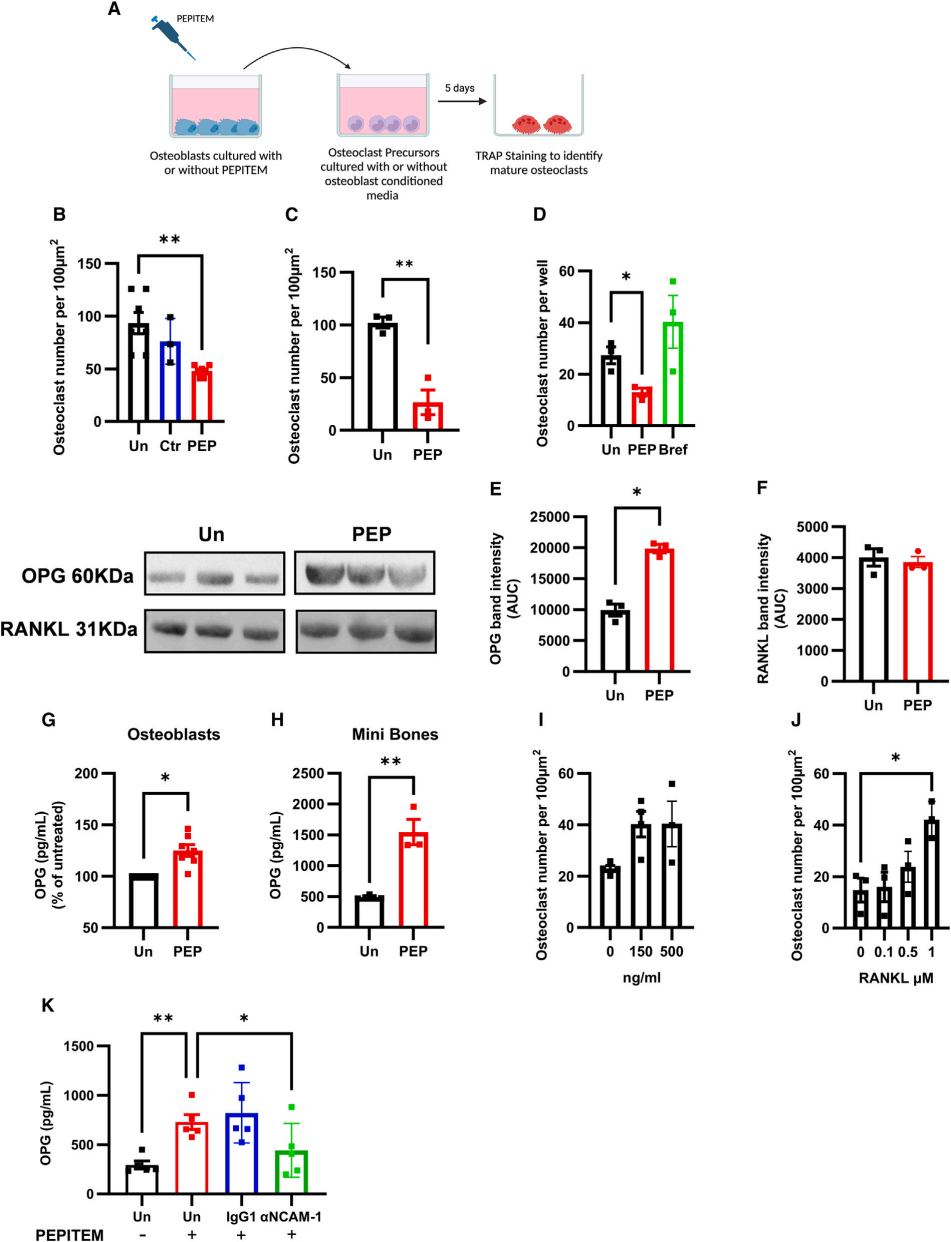
PEPITEM induces osteoblasts to release a soluble mediator, which inhibits osteoclast function For a Figure360 author presentation of Figure 6, see https://doi.org/10.1016/j.xcrm.2024.101574. (A–K) Schematic representation of protocol. (B and E–G) Murine macrophage-like RAW264.7 cell line or (C and D) human peripheral blood monocytes were cultured with conditioned media from (B–G and K) primary murine calvarial osteoblasts, or (H) mini-bones cultured alone (untreated, Un, black), with a control peptide (Ctr, blue), PEPITEM either alone (PEP, red) or (D) in combination with Brefeldin A (Bref, green), (I) anti-OPG antibody, (J) RANKL, or (K) anti-NCAM-1 antibody. (B–D, I, and J) Number of TRAP-positive osteoclasts expressed per 100 mm^2^. Data are mean ± SEM from *n* = 3–4, or (B) *n* = 7 independent experiments. ∗*p* < 0.05 and ∗∗*p* < 0.01 by (B, D, and I) Dunnett’s post-test. (E, G, H, and K) OPG and (F) RANKL protein in supernatants expressed as (E and F) band intensity (AUC), (G) percentage of untreated calvarial osteoblasts, (H) pg/mL for human mini-bone organoids or (K) calvarial osteoblasts. Data are mean ± SEM from *n* = 3–4, (K) *n* = 5, or (G) *n* = 7 independent experiments. ∗*p* < 0.05 and ∗∗*p* < 0.01 by paired t test, (G) Wilcoxon, (H) unpaired t test, or (K) Dunnett’s post-test.

Osteoblasts can regulate osteoclastogenesis through paracrine signaling often through the RANKL-OPG axis. Osteoblasts secrete both RANKL and OPG, which have opposing functions—RANKL binds to its receptor (RANK) on monocytes to induce their differentiation to osteoclasts; while OPG acts as a decoy receptor for RANKL, inhibiting its binding to RANK and thus limiting osteoclast differentiation.^32^ Given this ability of osteoblasts to control osteoclastogenesis, we examined the possibility that the negative regulation of osteoclast function was due to production of a soluble agent released by osteoblasts in response to PEPITEM. To test this hypothesis, we collected the secretome/supernatant from PEPITEM-treated osteoblasts and transferred this onto osteoclast precursors (Figure 6A). Osteoclastogenesis in murine (Figure 6B) and human (Figure 6C) precursors was significantly inhibited in the presence of conditioned media from PEPITEM-treated osteoblasts, suggesting the presence of an inhibitory agent. When osteoblasts were treated with PEPITEM in the presence of Brefeldin A (a chemical inhibitor of the golgi), they were no longer able to generate the osteoclast inhibitory agent(s) (Figure 6D). Importantly, as PEPITEM does not influence osteoclasts directly (Figures 5C–5E), these data strongly indicate that osteoblasts are releasing a soluble mediator in response to PEPITEM that inhibits osteoclast function.

Given this, we next assessed OPG and RANKL levels in the conditioned media released from osteoblasts following PEPITEM treatment. Significantly more OPG protein was detected in supernatants from PEPITEM-treated osteoblasts or 3-D human mini-bones organoids compared with untreated controls as assessed by western blot (Figure 6E) and ELISA (Figures 6G and 6H). By contrast, the amount of osteoblast-derived RANKL protein remained unchanged following PEPITEM treatment (Figure 6F). To ascertain whether OPG was truly the osteoblast agent responsible for inhibiting osteoclastogenesis, we used an OPG neutralizing antibody to deplete OPG from the PEPITEM-treated osteoblast conditioned media prior to adding it to the osteoclast precursors. Depleting OPG from the osteoblast conditioned media reversed the effects of PEPITEM on osteoclastogenesis, such that numbers of osteoclasts significantly increased (Figure 6I). To further confirm these observations, we undertook a competition assay, adding increasing concentrations of RANKL to the OPG-rich PEPITEM-treated osteoblast conditioned media. As expected, RANKL was able to outcompete PEPITEM-induced OPG, such that at 500 ng/mL RANKL the indirect inhibitory effects of PEPITEM on osteoclastogenesis were lost (Figure 6J). Finally, we assessed whether blocking NCAM-1 signaling impacted PEPITEM-induced OPG in osteoblasts. Significantly lower OPG concentrations were detected in conditioned media from anti-NCAM-1+PEPITEM-treated osteoblasts compared with those treated with PEPITEM in the presence of IgG1 control (Figure 6K). Collectively these data indicate that in response to PEPITEM signaling through NCAM-1, osteoblasts release OPG, which in turn negatively regulate osteoclast numbers, leading to an overall reduction in bone resorption and increase in bone density.

### PEPITEM reverses bone loss in age-related musculoskeletal diseases

Osteoporosis is the most commonly occurring MSK condition, resulting in a net loss of cancellous bone and increased risk of fracture. To date, most osteoporosis medications target the activity of the osteoclast (e.g., bisphosphates, denosumab) to prevent further bone loss, but are unable to induce bone repair. Recent advances have enabled the development of anabolic agents, such as teriparatide (a parathyroid hormone mimic) and romosozumab, that can induce new bone formation but have limitations clinically—teriparatide is only effective for 24 months^33^ and romosozumab has been associated with cardiovascular events.^34^ Therefore, new drugs targeting the endogenous repair processes elicited by osteoblasts are urgently required. Given the pro-osteoblastogenic actions of PEPITEM revealed above, we sought to investigate the therapeutic efficacy of PEPITEM in a model of excessive bone loss.^35^

**Figure 7:**
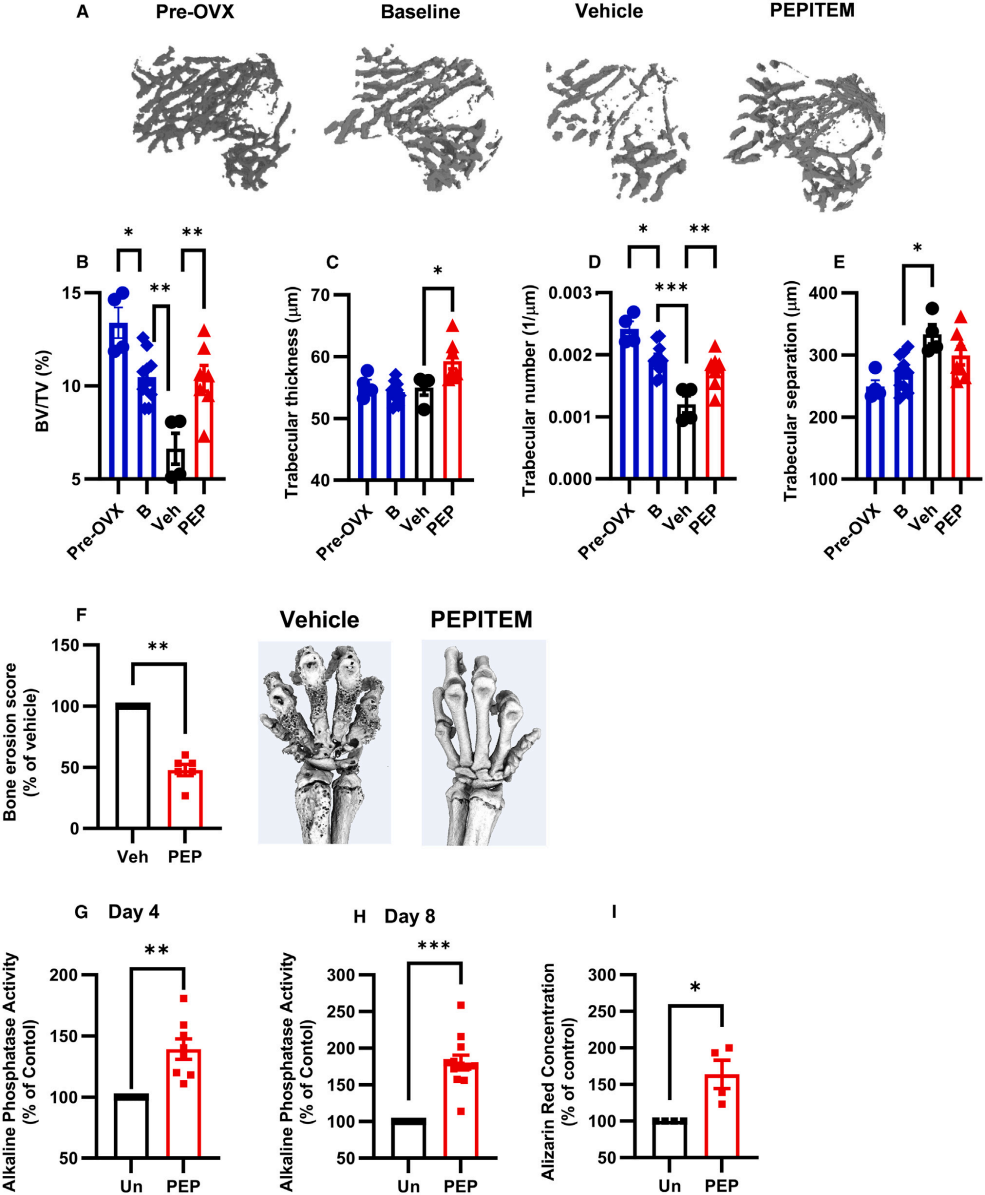
PEPITEM reverses bone loss related to musculoskeletal diseases (A–E) Osteoporosis was induced for 2 weeks prior to mice being injected with vehicle control (Veh, black, *n* = 4) or PEPITEM-PEG (PEP, red, *n* = 8) for a further 2 weeks. Baseline samples were analyzed 2 weeks post-surgery before treatment started (B, blue, *n* = 10). (A) Representative 3-D microCT renders of trabecular bone. (B) Percentage trabecular bone volume (BV/TV). (C) Trabecular thickness in μm. (D) Trabecular number per μm. (E) Trabecular separation in μm. In B-E, ANOVA showed significant effect of time and treatment on all bone parameters, *p* < 0.05. (F) Arthritis was induced, and mice were injected with vehicle control (Veh, black, *n* = 5) or PEPITEM-PEG (PEP, red, *n* = 5) for 2 weeks, prior to analysis by microCT. Bone erosion score expressed as a percentage of vehicle control. (G–I) Alkaline phosphatase activity at day (G) 4 (*n* = 8) or (H) 8 (*n* = 12) or (I) alizarin red concentration at day 18 (*n* = 4) normalized to percentage of untreated control for osteoblasts from aged patients left untreated (Un, black) or treated with PEPITEM (PEP, red). Data are mean ± SEM. ∗*p* < 0.05, ∗∗*p* < 0.01, and ∗∗∗*p* < 0.001 by (B–E) Dunnett’s post-test compared with PBS control treatment at 4 weeks, (F–H) Wilcoxon, or (I) Mann-Whitney U.

Osteoporosis onset is linked with menopause and the symptoms can be replicated *in vivo* upon the removal of the ovaries, where bone loss is evident as early as 2 weeks post-surgery (Fig S7). PEPITEM therapy limited the amount of trabecular bone loss triggered by ovariectomy (Figure 7A) with comparable levels of bone volume density (BV/TV), trabecular number, and thickness in the tibia of PEPITEM-treated mice at 4 weeks and those at the start of the 2-week therapy regimen (Figures 7B–7D). In contrast, there was a significant reduction in all these parameters in mice treated with vehicle control. As expected, we observed the reverse changes in trabecular separation (Figure 7E), indicating that PEPITEM therapy maintained a higher level of interconnectivity within the trabecular network during the onset of osteoporosis. Similar findings were seen in an inflammatory model of bone erosion, where PEPITEM treatment significantly reduced bone damage in arthritic mice when compared with vehicle-treated animals (Figure 7F). Using osteoblasts from aged patients (Table S5), we asked whether these cells were responsive to PEPITEM therapy. Crucially, PEPITEM significantly increased osteoblast maturation (Figures 7G and 7H) and mineralization (Figure 7I). These data highlight that PEPITEM could be used as an alternative and early clinical intervention to reverse the impact of age-related MSK diseases.

## Discussion

Dysregulation of bone remodeling underpins numerous MSK disorders resulting in substantive health care and socioeconomic costs through the permanent loss of function, pain, increased risk of fracture, and frailty in patients. Here we have described that the endogenous peptide, PEPITEM, regulates anabolic and catabolic activity in the bone. Specifically, PEPITEM acts directly on osteoblasts to induce new bone formation to increase bone strength. Simultaneously, PEPITEM triggers the release of the anti-osteoclastogenic regulator, OPG, from osteoblasts to limit bone resorption. Mechanistically, PEPITEM operates through a known bone regulating pathway—inducing rapid translocation of β-catenin to the nucleus to alter gene expression within osteoblasts (Figure 6). Crucially, PEPITEM therapy halted any further bone loss following ovariectomy and was effective at inducing bone formation by osteoblasts isolated from aged donors with osteoarthritis. Of note, we observed no sexual dimorphism in the response of cells, mice, or patients to PEPITEM. The dual bioactivity of PEPITEM is rare within the current portfolio of drugs available for MSK diseases, shared only by romosozumab, thus offering an alternative approach that promotes bone formation and repair while simultaneously redressing the imbalance in bone turnover.

PEPITEM can be endogenously cleaved from its parent protein 14-3-3ξ.^15^ Of the seven members of the 14-3-3 family, only two have been previously postulated to play a role in bone development and remodeling. Using short hairpin RNA to inhibit 14-3-3β significantly reduced bone growth and osteoblast number *ex vivo* and reduced MSC osteoblastogenesis *in vitro*, indicating it has an anti-anabolic role within the bone.^13^ By contrast, small interfering RNA (siRNA) knockdown of 14-3-3ξ (and thus PEPITEM) significantly reduced osteoblastogenesis *in vitro*—with osteoblast precursors showing diminished expression of maturation markers (*runx2, alp, col1a1, bmp*) resulting in reduced maturation/alkaline phosphatase activity and mineral production.^14^ However, and in contrast to our findings with PEPITEM, overexpression of 14-3-3ξ had no impact on BV/TV or trabecular number in resting Balb/c mice over the 6-week time frame of the experiment.^14^ Possible explanations for these differences include the use of different strains of mice; the time point chosen for the analysis (2 vs. 6 weeks); the type and bioactivity of treatment (bioactive peptide vs. microRNA antagomir induced up-regulation of 14-3-3ξ and required subsequent proteolytic cleavage). Overexpression of 14-3-3ξ was able to partially reverse ovariectomy-induced loss of cancellous bone density and trabecular number over 6 weeks of treatment in Balb/c mice.^14^ Importantly, these changes were comparable to those we observed with only 2 weeks of PEPITEM therapy, suggesting the pre-processed peptide represents a better therapeutic option. This is further supported by the ubiquitous expression pattern and multitude of cellular responses elicited by 14-3-3ξ and its up-regulation in various cancers (reviewed by Obsilova and Obsil^11^), which collectively make it a poor candidate for drug development. Conversely, PEPITEM circulates within serum of healthy individuals and its levels have been shown to decline with age,^15^ making it a strong therapeutic candidate and putative causative factor in age-related bone loss.

NCAM-1 is transiently expressed within the bone: its expression is maximal in osteoblast precursors,^24^ diminishing with maturation or long-term culture^23^ so that it is lost entirely in mature osteocytes.^24^ Indeed, osteoblast differentiation was significantly inhibited *in vitro* when NCAM-1 was silenced using either plasmid-based transfection of MC3T3-E1 cells or use of MSC from NCAM-1 knockout mice.^21^ When NCAM-1 was silenced, osteoblasts exhibited reduced total β-catenin and PI-3K protein^21^ and activation of Wnt/β-catenin in the absence of NCAM-1 restored osteoblastogenesis.^21^ β-catenin is essential for the differentiation^26^ and survival^27^ of osteoblasts and its signaling role in the bone remodeling process is relatively well understood. Sclerostin binds to the Wnt receptor LRP5/6, inhibiting Wnt from binding to its receptor and therefore preventing signaling via the canonical Wnt signaling pathway. An absence of signaling via this pathway causes β-catenin to be targeted for ubiquitination and subsequent proteasomal degradation. Romosozumab (anti-sclerostin antibody) exerts its bone-anabolic effect by preventing sclerostin binding to the LRP5/6, allowing Wnt to bind, which inhibits ubiquitination of β-catenin and enables its translocation to the nucleus where it induces transcription of *RUNX2* and *OPG* to cause osteoblast maturation. Here, we show that PEPITEM rapidly induced β-catenin activation and nuclear translocation to enhance the expression of genes involved in osteoblast differentiation, including *col1a1*, as well as driving increased secretion of OPG—a known Wnt target.^36^ Our *in silico* analysis predicts binding of PEPITEM to the FIII domain of NCAM-1, a region in which FGFR1 has been shown to bind and signal.^37^^,^^38^^,^^39^ FGFR1 is important in regulating osteoblastogenesis: deficiency in osteoblast precursors results in maintenance of stem-like properties and blockade of differentiation and mineral deposition.^40^ However, within mature osteoblasts^40^ and osteocytes lack of FGFR1 drives bone formation by increasing the amount of active β-catenin, leading to increased OPG release.^41^ Of note, we observe similar findings in response to PEPITEM, with increased active β-catenin within 15 min. Thus, it is possible that PEPITEM is binding to NCAM-1 directly or indirectly inhibits FGFR1 activation, driving increased β-catenin activity, osteoblast maturation, and mineralization, and OPG secretion resulting in enhanced bone formation and reduced osteoclastogenesis. However, further studies, such as site-directed mutagenesis or inhibition of the NCAM-1 FIII domain (FGFR1 binding site) are now required to conclusively prove this.

Of note, NCAM-1 is not the only PEPITEM-binding partner within the bone. Cadherin-15 was originally identified as the PEPITEM receptor and is expressed by osteoblasts and osteocytes^42^; however, our *in vitro* data indicate no role for cadherin-15 in mediating the activities of PEPITEM within the bone. Our co-immunoprecipitation studies revealed an additional nine potential binding partners with reported roles in bone homeostasis, of which eight were intracellular proteins with enzyme activity, roles in signaling pathways, or vesicle/ER trafficking of proteins. Of these intracellular proteins, nucleoredoxin, sec31A, and catenin delta-1 (p120) had high predicted interaction scores (pLTTD >90) with PEPITEM when analyzed using AlphaFold coupled with ChimeraX (data not shown), suggesting the possibility the PEPITEM influences intracellular responses in osteoblasts. Focusing on surface-expressed binding partners, EHD2 was also identified as a potential PEPITEM interactor. Little is known about the role of EHD2 in the bone, although its expression has been reported in osteoblastic-cell lines where it is thought to contribute to tumor suppression in the context of osteosarcoma.^43^ In other mesenchymal stem cell-derived cells (e.g., adipocytes), EHD2 is known to stabilize calveolin-1 to regulate lipid uptake^44^ through the protein kinase A-cAMP signaling pathway.^45^ There is no evidence to date to indicate that EHD2 responses are linked to the β-catenin translocation we observe upon PEPITEM treatment but further studies, including surface plasmon resonance, are necessary to reveal whether PEPITEM can interact with these other potential binding partners, and the functional consequence of such interactions, if they occur.

The balance between bone formation and bone resorption is largely controlled by the amount of RANKL and OPG produced by cells in the bone. Indeed, pharmaceutical companies have taken advantage of manipulating the RANKL-OPG ratio by developing biologics that either block RANKL (denosumab—anti-RANKL antibody) or reduce its endogenous production (romosozumab). By contrast, PEPITEM acts to enhance the production of the endogenous anti-osteoclastogenic factor, OPG, by osteoblasts without impacting RANKL levels—thereby swinging the balance in favor of bone formation without impacting the ability of osteoclasts to resorb regions of damaged or weak bone tissue via normal bone remodeling.

Bisphosphates are the current standard of care for osteoporosis, yet poorly managed long-term use is associated with increased incidence of microfractures and atypical femur fractures due to the absence of bone remodeling. Of the other remaining treatment options, only romosozumab has dual bioactivity—impacting both osteoblast and osteoclast function, as described for PEPITEM. Comparing mode of actions, PEPITEM is endogenously produced and acts directly on osteoblasts to stimulate osteoblastogenesis, while the humanized monoclonal antibody romosozumab works by removing the osteoblast inhibitor sclerostin to facilitate bone formation. Furthermore, PEPITEM stimulates osteoblast release of the endogenous inhibitor OPG, while romosozumab limits RANKL release to reduce bone resorption. The early data on the clinical efficacy of romosozumab appears exciting; however, this only appears effective over a relatively short term (maximum duration of treatment is 12 months) with reports of increased cardiovascular events in patients.^34^ As an endogenous osteogenic peptide with the capacity to regulate osteoblast-osteoclast coupling in health and disease, PEPITEM offers the real possibility for maintenance or restoration of bone homeostasis over the long-term to prevent osteoporosis and fragility fractures. For this to be realized, prolonged treatment protocols are now required in a variety of bone disease models to ascertain the quality of the bone formed in response to PEPITEM therapy.

### Conclusions

PEPITEM exerts osteogenic properties—whereby its osteogenic activity regulates osteoblast-osteoclast coupling leading to enhanced bone formation and moderating bone resorption in health, with age, and in disease (osteoporosis and rheumatoid arthritis) to redress the imbalance in bone turnover. Further studies are now urgently required to ascertain the therapeutic potential of PEPITEM or a PEPITEM-based agent in the clinical management of patients with excessive bone loss.

### Limitations of the study

This study revealed 10 potential PEPITEM-binding partners on osteoblasts (listed in Table S1). Of these, we further investigated the role of one (NCAM-1) *in vitro* and *ex vivo*, but not *in vivo* in the murine models. Our *in silico* modeling provides a predicted analysis for protein-protein interactions, but it is important to consider surface plasma resonance and X-ray crystallography are the gold standard techniques to determine receptor-ligand interactions and these were not performed in the current study. The proposed molecular mechanism does not directly link NCAM-1 signaling to β-catenin activation and translocation—site-directed mutagenesis-type studies are needed for this. Finally, the use of PEPITEM therapy *in vivo* requires further pharmacokinetic and pharmacodynamic studies in a broader range of applications (e.g., fracture models, immobilization studies) in mice and other species, prior to translation into clinical use.

## STAR★Methods

### Key resources table

**Table:** 

REAGENT or RESOURCE	SOURCE	IDENTIFIER	
**Antibodies**			

Anti-Cadherin-15 Antibody, clone: 12G4	Sigma-Aldrich	Cat: 05-852; RRID: AB_390133	
Anti-NCAM-1 Antibody, clone: MEM-188	Thermo Fisher Scientific	Cat: MA1-19129; RRID: AB_1073124	
Rabbit IgG1 Isotype Control Antibody	Invitrogen	Cat: 02-6102; RRID: AB_2532938	
Anti-RANKL Antibody, clone: 3F2E1	Proteintech	Cat: 66610-1-Ig; RRID: AB_2881970	
Anti-OPG polyclonal Antibody	Bio-Techne	Cat: AF459-SP; RRID: AB_3553	
Anti-β-actin polyclonal Antibody	Sigma-Aldrich	Cat: A2066; RRID: AB_476693	
Goat Anti-Mouse IgG HRP-conjugated Antibody	Bio-Rad	Cat: 1706516; RRID: AB_2921252	
Goat Anti-Rabbit IgG HRP-conjugated Antibody	Merck Millipore	Cat: 401393; RRID: AB_437797	
Anti-β-catenin polyclonal Antibody	Merck Millipore	Cat: 06-734; RRID: AB_310231	
Donkey Anti-Goat IgG 647-conjugated Antibody	Thermo Fisher Scientific	Cat: A21447; RRID: AB_2535864	
Goat anti-Rat IgG 546-conjugated Antibody	Thermo Fisher Scientific	Cat: A11081; RRID: AB_2534125	
Goat anti-Rabbit IgG 488-conjugated Antibody	Thermo Fisher Scientific	Cat: A11034; RRID: AB_2576217	
Anti-CD56 (NCAM) Antibody, clone: 5.1H11	Thermo Fisher Scientific	Cat: AB5032; RRID: AB_2291692	
Anti-RUNX2 Antibody, clone: 232902	Bio-Techne	Cat: MAB2006-SP; RRID AB_2184526	
Rabbit Non-phospho (Active) β-Catenin (Ser33/37/Thr41) (D13A1) Antibody	Cell Signaling	Cat: 8814T; RRID AB_11127203	

**Biological samples**			

Murine Tibias and Spines	C57BL/6J male mice	N/A	
Murine Tibias and Spines	Ovariectomy female C57BL/6J mice	N/A	
Embryonic metatarsals	C57BL/6J embryonic (E14.5) mice	N/A	
Hind Limbs	Collagen induced arthritis male DBA-1 mice	N/A	
Human bone chips	Royal Orthopedic Hospital, Birmingham	N/A	
Small dentine disks	Gift from Prof James Edwards, University of Oxford	N/A	

**Chemicals, peptides, and recombinant proteins**			

PEPITEM-PEG (SVTEQGAELSNEER- PEG(352)-Amide)	Cambridge Bioscience	N/A	
PEPITEM (SVTEQGAELSNEER)	Cambridge Bioscience	N/A	
Control peptide (EQAERYDDMAACMK)	Cambridge Bioscience	N/A	
biotinylated PEPITEM (Biotin-SVTEQGAELSNEER)	Cambridge Bioscience	N/A	
biotinylated control peptide (Biotin-EQAERYDDMAACMK)	Cambridge Bioscience	N/A	
Complete Freund’s Adjuvant	Sigma-Aldrich	Cat: F5881	
Bovine collagen II	Gift from Prof Richard Williams, University of Oxford, UK	N/A	
Sphingosine-1-phosphate	Sigma-Aldrich	Cat: S9666	
Recombinant Mouse RANKL	Abcam	Cat: Ab129136	
Recombinant Human M-CSF	R&D systems	Cat: 216-MC-005/CF	
Recombinant Human RANKL	R&D systems	Cat: 390-TN-010/CF	
Zoledronic acid hydrate	Cambridge Biosciences	Cat: CAY14984	
Recombinant Mouse FGFR1	Sino Biological	Cat: 50186-M02H	

**Critical commercial assays**			

Alizarin Red S Staining Quantification Kit	Caltag+Medsystems	Cat: 8678	
BCA assay	Thermo Fisher Scientific	Cat: 23225	
EasySep™ Human Monocyte Isolation Kit	STEMCELL Technologies	Cat: 19359	
Osteo Assay Surface Multiple Well Plate	Corning	Cat: CLS3987	
Mouse Osteoprotegerin Quantikine ELISA Kit	R&D systems	Cat: MOP00	
Human Osteoprotegerin DuoSet ELISA Kit	R&D systems	Cat: DY805	
Mouse RANKL Quantikine ELISA Kit	R&D systems	Cat: MTR00	
Rat/Mouse Total procollagen type-1 N terminal propeptide (P1NP) EIA	RatLaps	Cat: AC-33F1	
RNeasy Kit	Qiagen	Cat: 74104	
High-Capacity cDNA Reverse Transcription Kit	Applied Biosystems	Cat: 10400745	

**Deposited data**			

Bulk RNAseq	SRA	http://www.ncbi.nlm.nih.gov/bioproject/1099588	

**Experimental models: Cell lines**			

Murine calvarial osteoblasts (C.Ob)	C57BL/6J	N/A	
ST2	Gift from Prof James Edwards, University of Oxford	N/A	
Primary Human Osteoblasts	Royal Orthopedic Hospital, Birmingham	N/A	
hFOb 1.19	ATCC	CRL-3602	
Primary human bone-derived mesenchymal stem cells	Lonza	C-14090	
Murine bone marrow osteoclast precursors	C57BL/6 WT	N/A	
RAW264.7	ATCC	TIB-71	
Human Monocytes	healthy volunteers	N/A	
C2C12	ATCC	CRL-1772	

**Experimental models: Organisms/strains**			

C57BL/6J male mice	Charles River	N/A	
Ovariectomy female C57BL/6J mice	Charles River or Envigo	N/A	
Collagen induced arthritis male DBA-1 mice	Charles River or Envigo	N/A	

**Oligonucleotides**			

Ncam1 (Mm01149710_m1)	Thermo Fisher Scientific	Cat: 4331182	
Alpl (Mm00475834_m1)	Thermo Fisher Scientific	Cat: 4331182	
Cdh15 (Mm00483191_m1)	Thermo Fisher Scientific	Cat: 4331182	
CDH15 (Hs00170504_m1)	Thermo Fisher Scientific	Cat: 4331182	
ACP5 (Hs00356261_m1)	Thermo Fisher Scientific	Cat: 4331182	
ACP6 (Hs00212563_m1)	Thermo Fisher Scientific	Cat: 4331182	
CTSK (Hs00166156_m1)	Thermo Fisher Scientific	Cat: 4331182	
MRS1 (Hs00234007_m1)	Thermo Fisher Scientific	Cat: 4331182	
Col1a1 (Mm00801666_g1)	Thermo Fisher Scientific	Cat: 4331182	

**Software and algorithms**			

NRecon 1.6.1.5	Bruker	N/A	
DataViewer	Bruker	https://www.bruker.com/en/products-and-solutions/preclinical-imaging/micro-ct/3d-suite-software.html	
CTAn v1.12	Bruker	https://www.bruker.com/en/products-and-solutions/preclinical-imaging/micro-ct/3d-suite-software.html	
Osteomeasure	Osteometrics	https://www.osteometrics.com/	
ImageJ	National Institute for Health	https://imagej.nih.gov/ij/	
ImageJ FIJI	ImageJ	https://imagej.net/software/fiji/downloads	
AlphaFold2_multimer	Google colab	https://colab.research.google.com/github/sokrypton/ColabFold/blob/main/AlphaFold2.ipynb#scrollTo=mbaIO9pWjaN0	
Gen5	BioTek	N/A	
Uniprot database using Protein Discovery 2.2	Thermo Fisher Scientific	N/A	
Sequest HT algorithm	Thermo Fisher Scientific	N/A	
ChimeraX	UCSF	https://www.cgl.ucsf.edu/chimerax/	
Zen Blue software	ZEISS	N/A	
FlowJo	BD Life Sciences	N/A	

**Other**			

Skyscan 1172 micro-CT scanner	Bruker	N/A	
ElectroForce® BioDynamic® 5500	Bose	N/A	
ZEISS Axioscan Z1	ZEISS	N/A	
Synergy HT plate reader	BioTek	N/A	
Cytation 5 microscope	Biotek	N/A	
170V current PowerPac Basic Power supply	Bio-Rad	Cat: 1645050	
Trans-Blot turbo transfer system	BioRad	Cat: 1704150	
ChemiDOC MP Imaging System	BioRad	N/A	
100V current PowerPac Basic Power supply	Bio-Rad	Cat: 1645050	
UltiMate® 3000 HPLC series	Dionex	N/A	
QExactive HF Orbitrap mass spectrometer	Thermo Fisher Scientific	N/A	
Triversa Nanomate nanospray source	Advion Biosciences	N/A	
LSM 780 confocal microscope	ZEISS	N/A	
LightCycler 480	Roche	Cat: 04729749001	

### Resource availability

#### Lead contact

Further information and requests for resources and reagents should be directed to and will be fulfilled by the lead contact, Helen McGettrick (h.m.mcgettrick@bham.ac.uk).

#### Materials availability

This study did not generate unique reagents. Key resources are outlined in the resources table.

#### Data and code availability

• Bulk RNA-seq data have been deposited at SRA (http://www.ncbi.nlm.nih.gov/bioproject/1099588) and are publicly available as of the date of publication. Accession numbers are listed in the key resources table.
• This paper does not report original code.
• Any additional information required to reanalyze the data reported in this paper is available from the lead contact upon request.

### Experimental model and study participant details

#### Animals studies

Mice were purchased from Charles River or Envigo, and were maintained in a specific pathogen free facility, with free access to food and water. Environmental conditions were: 21 ± 2°C, 55 ± 10% relative humidity and a 12 h light-dark cycle.

Eight-week-old, male, C57Bl/6J resting wild type (WT) mice were given daily intra-peritoneal (IP) injections of equivalent volume of vehicle control (PBS), or pegylated PEPITEM (300 μg, sequence SVTEQGAELSNEER- PEG(352)-Amide; both from Cambridge Research Biochemicals Limited; Cambridge, UK) for 14 or 28 days. On day 8 and day 12, mice were given 20 mg/kg calcein (Sigma-Aldrich) by IP injection.

Alternatively, 12-week-old, female C57Bl/6J underwent ovariectomy. Fourteen days later, mice were culled to acquire baseline measurements or given daily IP injections of either vehicle control (PBS) or PEPITEM-PEG (300 μg) for 14 days. Ovariectomy is only possible on female mice, and the effects of PEPITEM on male mice during osteoporosis-related bone loss were not explored.

Alternatively, 8-week-old, male DBA-1 were administered s.c. injection of bovine collagen II (CII, 100 μg, gift from Prof Richard Williams, University of Oxford, UK) 1:1 with complete Freund’s adjuvant, followed by boost at day 21 with CII in incomplete Freund’s adjuvant. Between days 21–35 mice were given daily IP injections of either vehicle control (PBS) or PEPITEM-PEG (300 μg).

Following treatment, mice were culled and bones from the hind limbs and spine were dissected, cleaned of muscle and fat before fixation in 10% neutral-buffered formalin (4% v/v formaldehyde in phosphate buffered saline - PFA) for 24 h and stored in 70% ethanol at 2-8°C until analysis.

In all experiments, littermates were randomly assigned to experimental groups within the same cages.

Animal studies were regulated by the Animals (Scientific Procedures) Act 1986 of the United Kingdom and performed under Personal Project Licence (PE5985209) at the Biomedical Services Unit, University of Birmingham, which holds a section 2C Establishment Licence. Approval was granted by the University of Birmingham’s Animal Welfare and Ethical Review Body and all ethical guidelines were adhered to whilst carrying out this study.

#### Human participants

Primary human osteoblasts were isolated from aged (between 55 and 80 years old) predominantly female patients undergoing joint replacement surgery at the Royal Orthopedic Hospital, Birmingham (Table S5). The skewing of participants sex to females is equivalent to osteoporosis incidence. Power calculations based on preliminary data indicated 10 participants per group per analysis were required. Sample size used is recorded within the legend for Figure 7. In all experiments utilising human samples, the same patient samples were included in all conditions. The study was conducted in compliance with the Declaration of Helsinki. All human samples were obtained with written, informed consent and approval from the Human Biomaterial Resource Center (Birmingham, UK), South East Scotland Research Ethics Committee (16/SS/0172) or University of Birmingham Local Ethical Review Committee.

#### Cell lines and primary cell cultures

##### Osteoblasts

Primary murine calvarial osteoblasts (C.Ob) were isolated from male and female postnatal day 3–5 C57BL/6J WT mice as previously described^39^ and cultured in basal murine osteoblast media [α-MEM, supplemented with 2 mM L-glutamine, 100 μg/mL streptomycin, 100U/ml penicillin (all from Sigma-Aldrich) and 10% fetal bovine serum (Biosera)], prior to use at passage (P)1. Gene expression for specific osteoblast progenitor and maturation markers, along with capacity to differentiate into osteoblasts and form mineral were used to authenticate the cell lineage.

Murine osteoblast precursor stromal P1 cell line (ST2) were cultured in basal osteoblast murine media and used before P9.

Primary human osteoblasts were isolated from aged patients undergoing joint replacement surgery at the Royal Orthopedic Hospital, Birmingham (Table S5) using the outgrowth method from bone chips as previously described^40^ and cultured in basal human osteoblast media [DMEM, 2 mM L-glutamine, 100 μg/mL streptomycin, 100 U/ml penicillin, 1% non-essential amino acid solution, 2 mM β-glycerol phosphate, 50 μg/mL L-ascorbic acid (all from Sigma-Aldrich) and 10% fetal bovine serum (Biosera)], prior to use at P1-4. Gene expression for specific osteoblast progenitor and maturation markers, along with capacity to differentiate into osteoblasts and form mineral were used to authenticate the cell lineage.

Human osteoblast cell line (hFOb 1.19) were cultured in DMEM/F-12, no phenol red (Fisher scientific, cat; 21041025) supplemented with 10% FBS and 0.3 mg/mL G418 (Sigma-Aldrich, cat; 4727878001) at 35.5°C for 3 days, before moving cells to 37°C and culturing in osteogenic media (basal culture media plus 10-8M menadione and 100 μg/ml ascorbic acid (all from Sigma-Aldrich).

Primary human bone marrow-derived mesenchymal stem cells (MSC) from healthy donors were purchased from Lonza Ltd (Basel, Switzerland) at P2 and expanded/cultured in MSCGM Bulletkit (Lonza), prior to use at P5. Cells were plated (3 x 10^4^) in 6 well plates or 1.5 x 10^4^ in 12 well plates and allowed to adhere for 24 h before changing to osteogenic media (Lonza, cat: PT-3002) with media changes every 3 days.

##### Osteoclasts

Murine bone marrow osteoclast precursors were isolated from the hindlimb tibias and femurs of 8-week-old male and female C57BL/6J WT mice by centrifugation at 10,000 g for 15 s in murine basal osteoclast media.^46^ The pellet was dispersed using a 25G needle before addition of murine basal osteoclast media and filtration through a 70 μm pore filter. Gene expression for specific osteoclast progenitor and maturation markers, along with capacity to differentiate into osteoclasts and resorb mineral were used to authenticate the cell lineage.

Murine macrophage-like cell line RAW264.7 (TIB-71; ATCC) were cultured in basal osteoclast media.

Primary human peripheral mononuclear cells (PBMC) were isolated from the blood of male and female healthy volunteers as previously described.^47^ Monocytes were negatively selected from PBMC resuspended in MACs buffer using EasySepTM Human Monocyte Isolation Kit, as per manufacturer’s instructions (Stem Cell, Cambridge, UK).

### Induction of myocyte differentiation

Immortalised murine myoblast cell line C2C12 (CRL-1772, ATCC) were cultured in DMEM supplemented with 2 mM L-glutamine, 100 μg/mL streptomycin and 100 U/ml penicillin (all from Sigma-Aldrich) for 24 h, prior to differentiation in DMEM supplemented with 2% horse serum (Sigma-Aldrich) for 8 days.

### Method details

#### microCT analysis

Limbs and spines were placed vertically into the Skyscan 1172 micro-CT scanner (Bruker; Kontich, Belgium) and imaged every 0.45° as previously described.^37^ Images were reconstructed using NRecon 1.6.1.5 (Bruker) by two independent researchers and subsequently analyzed blinded and independently. Briefly, scanning misalignment was calculated and automatic compensation was applied, followed by a beam hardening correction of 20% and a ring artifact correction of 4. Cross-sectional images were reconstructed using the Feldkamp algorithm. Spinal cross-sectional images were subsequently rotated to 3D coronal view in DataViewer (Bruker).

Trabecular and cortical bone parameters were calculated from cross-sectional images and 3D images generated using the CTAn v1.12 software (Computed Tomography Analyser; Bruker). Comparing CTAn images to original CT images allowed a manual global threshold that differentiated “real” bone from background noise to be applied. Regions of interest (ROIs) beginning at the distal-most point of the proximal growth plate were drawn around trabecular bone. 100 slices were included (total region of 1370μm) for tibiae and 14 (total region of 192 μm) for the spine. The “despeckle” CTAn plugin was used to remove white pixels from 3D space, then analyzed as 2D images or converted to a 3D mesh using a marching cube algorithm in CTAn, before meshes were visualised in MeshLab 1.3.2 (ISTI-CNR, Italy). Quantitative data was generated by the “3D analysis” plugin (CTAn) and percentage trabecular bone volume/tissue volume (BV/TV %); average (mean) trabecular number per μm as 1/μm; average (mean) trabecular thickness in μm and average (mean) trabecular separation in μm are shown. For the arthritis model, images were scored for degree of erosion (0 = normal, 1 = roughness; 2 = pitting or 3 = full thickness holes) and the extent of damage (0 = none, 1 = a few small areas; 2 = multiple small to medium sized areas or 3 = multiple medium to large sized areas). Data were presented as bone erosion as a percentage of the vehicle treated animals.

#### Mechanical testing of murine femurs

Femurs were cleaned of all muscle and tested to the point of failure in the 3-point bending test using the ElectroForce BioDynamic 5500 (Bose, ElectroForce Systems Group, Minnesota, USA) as previously described.^37^ A 22 N load cell was placed under the bone and set to descend at 1 mm/min. Force and displacement data were generated as displacement (mm) against force (N) measured every second. Data were expressed as bone stiffness (N/mm; gradient of the rising portion) and force at failure (N; maximum load supported by the bone).

#### Tissue analysis

Dynamic histomorphometry of calcein-labelled bone embedded in plastic was performed at the University of Sheffield by the Skelet.AL team (Sheffield, UK). Sagittal sections of the tibia (8 μm) were cut at 3 depths, 30 μm apart, and the endocortical bone surface was imaged using Osteomeasure software (Osteometrics, Georgia, USA). Six fields of view were taken along both the medial and lateral surfaces starting 0.33 mm from the growth plate, totalling a distance of 3.6 mm^38^. Bone formation rate normalised to bone surface perimeter (BFR/BS) was expressed as μm3/μm2/day and the length of double calcein labels normalised to total bone surface perimeter (dL.s/BS) was expressed as a percentage.

Osteoclast or osteoblast number in tibial bone formalin-fixed paraffin-embedded (FFPE) sections were analyzed by immunohistochemistry for tartrate-resistant acid phosphatase (TRAP) activity or haematoxylin and eosin (H&E) staining, respectively. Sections were deparaffinised using Xylene (VWR), followed by rehydration in decreasing concentrations of ethanol (100%, 90%, 80%, 70%) and finished with two washes with de-ionised water.

For H&E staining, deparaffinised slides underwent serial staining for haematoxylin (Pioneer Research Chemicals) and eosin (Pioneer Research Chemicals). Slides cleared in xylene for two 5 min incubation. Slides mounted in xylene-based mounting medium (Merck) and imaged using the ZEISS Axioscan Z1. Images analyzed using ImageJ by two independent researchers, where total bone surface starting 100 μm under the growth plate was measured and a ROI created. From this ROI area of bone which had osteoblasts present were measured, where an osteoblast was characterised as multiple cuboidal cells lining the bone. Osteoblast number presented as percentage bone osteoblast coverage (Ob.S/BS).

For analysis of osteoid deposition,^46^ rehydrated slides were stained with 0.04% Fast Green for 30 min before being rinsed in running water for 1 min. Sections were then stained with 0.1% sirius red dissolved in picric acid for a total of 1h. Finally, sections were rinsed in water, dehydrated in ethanol and cleared in xylene before being mounted with xylene-based mounting medium (Merck). Slides were imaged using the ZEISS Axioscan Z1 and analyzed using ImageJ by two independent researchers. Briefly, an ROI was created around trabecular bone below the growth plate and total trabecular bone surface area was measured. Surface area of red staining on the surface of the bone was then measured and presented as percentage of osteoid compared to trabecular bone surface area (OB/OS).

For TRAP staining deparaffinised slides were submerged for 30 min at 37°C in the TRAP staining solution [0.11 mM sodium acetate anhydrous (VWR), 0.074mM L-(+)-Tartaric acid (Fisher Scientific), 2% (v/v) glacial acetic acid supplemented with 1.6M Fast Red Violet LB salt and 2% (v/v) 58 mM Naphthol AS-MX (all from Sigma-Aldrich) diluted in 2- ethoxyethanol (Alfa Aesar, Lancashire, UK)]. Slides were mounted using immuno-mount (Fisher Scientific) and imaged using the ZEISS Axioscan Z1. Images were color separated in ImageJ using CIELAB color space (L∗A∗B∗), where L∗ (lightness) and B∗ (blue/yellow) were set as 0 to remove colors that are not red. Manual thresholding on A∗ (green/magenta) to limit visible spectrum to pink osteoclasts. Osteoclasts were counted manually using the ImageJ cell counter plugin, normalised to the area of the ROI, and presented as osteoclasts/mm^2^.

#### Osteoblast maturation and mineralisation assays

Osteoblasts were seeded at 10^4^ cells/well into 48 well plates and cultured in either basal media or osteoblastogenic media [murine/human basal media supplemented with 10 mM β-glycerol phosphate and 50 μg/mL L-ascorbic acid (Sigma-Aldrich)] for up to 21 days. In some cases, media was supplemented with either: 10 ng/mL PEPITEM (sequence: SVTEQGAELSNEER), 10 ng/mL control peptide (sequence: EQAERYDDMAACMK, both from Cambridge Bioscience), 10 μg/mL cadherin-15 agonist antibody (cat: 05–852; Sigma-Aldrich), 3 μg/mL anti-NCAM-1 antibody (clone MEM-188; Thermo Fisher), IgG1 isotype control antibodies (3 μg/mL, Invitrogen cat; 02–6102) or increasing concentrations of sphingosine-1-phosphate (S1P, Sigma-Aldrich). Supernatants were collected at various timepoints for analysis in secretome assays, by ELISA or western blot (see sections below). For western blot analysis, cells were serum starved for 24h prior to centrifugation of supernatants at 10000 rcf for 20 min. Supernatants were stored at −80°C until use.

Osteoblast maturation was assessed in technical triplicate by quantifying alkaline phosphatase activity. Briefly, cells were lysed in RIPA buffer (Sigma-Aldrich) for 30 min on ice, harvested using a cell scraper and centrifuged at 13,000g for 10 min. A 1:4 ratio of cell lysate to alkaline phosphatase yellow (pNPP) liquid substrate for ELISA (Sigma-Aldrich) were incubated for 45 min in the dark at 37°C with agitation (SciQuip, Shropshire, UK) before being quantified using a Synergy HT plate reader with absorbance set at 405 nm. Data are expressed as percentage of control (%).

Osteoblast mineralisation was assessed using the Alizarin Red S Staining Quantification Kit in technical duplicate and following manufacturer’s instructions (Caltag+Medsystems, Buckingham, UK). Entire wells were imaged using the Cytation 5 microscope and Gen5 software (both Biotek). The alizarin red stain was quantified by dye extraction as per manufacturer’s instructions and absorbance read using a Synergy HT plate reader with absorbance set at 405 nm. The concentration of alizarin red (mM) was quantified using a standard curve generated from known concentration standards and expressed as a percentage of the untreated control.

#### Metatarsal methods

Hind limbs from embryonic day (E)14.5–15.5 mice were removed, and metatarsals dissected under a dissection microscope in α-MEM (Sigma-Aldrich) diluted 1:13 with sterile PBS, plus 2 mg/mL BSA. The metatarsals were cultured in α-MEM, supplemented with 2 mM L-glutamine, 100 μg/mL streptomycin, 100 U/ml penicillin, 0.2% BSA, 5 μg/mL L-ascorbic acid and 1 mM β-glycerol phosphate (all from Sigma-Aldrich) for 5 days prior to the start of the experiment. Subsequently, metatarsals were treated ± PEPITEM alone, with IgG1 or anti-NCAM-1 antibody. Metatarsals were imaged every other day and data expressed as increase in mineralisation zone in μm.

#### Human self-structuring bone organoids

Three-dimensional self-structuring bone models (SSBM) containing hFOb 1.19 were created as previously described.^47^ SSBM were cultured in osteogenic media (DMEM/F-12, no phenol red with 10% FBS, 0.3 mg/mL G418, 10^−8^M Menadione and 100 μg/mL Ascorbic acid (as detailed above) at 37°C and 5% CO_2_ for 4 weeks prior to addition of treatment - with or without 10 ng/mL PEPITEM or control peptide for 8 days. Three days prior to analysis, constructs were moved into fresh plates with fresh media ± treatments to ensure that only encapsulated cells (and not populations growing outside of the construct on the tissue culture plastic) were included in the analysis. Conditioned media from the last 72 h of culture was collected and analyzed by OPG ELISA (see below).

#### Western blot

Cells were incubated in RIPA buffer (Sigma-Aldrich) for 30mins on ice, prior to storage at −80°C. Total protein was assessed using BCA assay (ThermoScientific) according to manufacturer’s guidelines. Samples were diluted 1:5 with SDS Laemmli buffer (Bio-Rad, cat-1610737), heated at 95°C for 10min prior to loading into 10% gels (Nu-Page, cat -NP0326BOX). Proteins were separated using a 170V current PowerPac Basic Power supply (cat-1645050) for 50 min, then the gel was transferred on to a PVDF membrane using the Trans Blot Turbo mini 0.2 μm (cat-1704156) and Trans-Blo turbo transfer system (all from BioRad).

Membranes were blocked with 5% milk (Marvel – Premier Foods, Ireland) diluted in PBS containing 1% TWEEN 20 (Sigma-Aldrich) for 1h under agitation, then incubated with the following primary antibodies diluted in 5% milk overnight at 4°C: anti-RANKL (1:3000, clone:3F2E1, Proteintech); anti-OPG polyclonal (1:3000, cat: AF459-SP Bio-techne); anti-NCAM- (1:1000 clone MEM-18, ThermoFisher Scientific); or anti-β-actin polyclonal (1:1000, cat:A2066, Sigma-Aldrich) or anti-none-phosphorylated β-catenin (1:10000, cat:8814T, Cell Signaling). Membranes were then washed with PBS-T and incubated with the relevant HRP-conjugated antibodies for 1h: mouse, 1:3000, cat:1706516, BioRad) or rabbit (1:5000, cat:401393, Millipore). Membranes were washed as described and treated with Clarity Western Peroxide Reagent and Clarity Western Luminol/Enhancer Reagent 1:1 (cat: 1705061) prior to imaging using ChemiDOC MP Imaging System (both from BioRad). Band intensity was measured using ImageJ FIJI software.

#### Co-immunoprecipitation and mass spectrometry

C.Ob were cultured in osteoblastogenic media for 24h then moved to FBS free osteoblastogenic media containing 0.1mg BSA ±3 μg/mL biotinylated PEPITEM (seq: Biotin-SVTEQGAELSNEER; Cambridge Bioscience) or biotinylated control peptide (seq: Biotin-EQAERYDDMAACMK) for 4 h at 4^o^C. Cells were washed in ice-cold PBS followed by incubation on ice in ice-cold Triton phosphate lysis buffer [50 mM Tris-HCL, pH 7.5; 150 mM NaCl; 1% Triton X-100; Protease and phosphatase inhibitor (Roche)] for 30 min. Lysates were centrifuged for 20 min at 600 g at 4°C, supernatant collected and stored at −80°C prior to use.

Pierce High-Capacity Streptavidin Chromatography Cartridges (ThermoFisher Scientific, cat: 87739) were equilibrated with Triton phosphate lysis buffer at a flow rate of 0.5–2 mL/min. Supernatants were then passed through the column at a flow rate of 0.5-1 mL/min and fractions were collected. Columns were washed ×5 with Triton phosphate lysis buffer prior to elution of bound proteins using 50% ACN with 1% trifluoroacetic acid (TFA; ThermoFisher). Fractions containing the eluted proteins were then dried in a vacuum centrifuge overnight. Samples resuspended in 8 M urea in 2% SDS and 20μL of the resuspended sample was added to 5 μL of SDS Laemmli buffer (Bio-Rad, cat: 1610737) prior to incubation at 95°C for 10min

Proteins in the samples were separated using a 4–12% Bis-Tris gels (Nu-Page, cat: NP0326BOX) using a 100V current PowerPac Basic Power supply (cat: 1645050; BioRad) for 90 min. Separation gels were stained with Bio-Safe Coomassie stain (cat: 1610786, BioRad) for 1 h room temperature with shaking, prior to washing ×4 in PBS. Regions with bands of high Coomassie blue staining were cut into 5 sections and stored in PBS. Samples were then sent for peptide identification, performed by the Mass Spectrometry Facility (School of Biosciences, University of Birmingham, UK).

Briefly, trypsin digested samples (≤10μg of protein) were incubated in 100 mM ammonium bicarbonate (pH 8) and 10 mM dithiothreitol for 30 min at 56^o^C. Cooled samples were incubated at room temperature in 50mM iodoacetamide for 30 min, prior to addition of trypsin gold (6 ng/μL; Promega, Southampton, Hampshire, UK) and incubation 37^o^C overnight. Peptide concentration and separation was performed using UltiMate 3000 HPLC series (Dionex, Sunnyvale, CA USA). Samples were added to precolumn (Thermo Scientific Acclaim PepMap 100 C18 HPLC Columns) and separated in Nano Series Standard Columns using a gradient of 3.2%–44% solvent B (0.1% formic acid in acetonitrile) for 30 min. Columns were washed with 90% mobile phase B, re-equilibrated with 3.2% mobile phase B and eluted directly (∼350 nL/min) via a Triversa Nanomate nanospray source (Advion Biosciences, NY) into a QExactive HF Orbitrap mass spectrometer (ThermoFisher Scientific).

Data were analyzed using Uniprot database using Protein Discovery 2.2 software, Sequest HT algorithm (Thermo Fisher). Precursor mass tolerance was 10 ppm and the MS/MS mass tolerance was 0.02 Da. Data were filtered with a false discovery rate of 0.01, and proteins with at least two high confidence peptides were accepted as a real hit. Quality control screening was performed on protein/peptide hits to remove expected contaminants and also hits that appeared in both control peptide and PEPITEM samples. The remaining potential PEPITEM binding partners were initially ranked using Sequest score, and subsequently filtered based on an isoelectric point score of 4–7 corresponding to proteins associated with the plasma membrane. Extensive literature analysis was performed on the top 10 binding partners looking for specific roles in bone biology.

#### NCAM expression by flow cytometry

Calvarial osteoblasts were grown in osteogenic media before being fixed prior to removal from well plates and subsequently resuspended in MACs buffer (PBS supplemented with, 0.5% EDTA, and 0.6% BSA (all from Sigma)). Cells were stained with anti-CD56 (NCAM) PE (1:50, clone 5.1H11, ThermoFisher Scientific). Samples were filtered and acquired using Fortessa-X20. Data were analyzed offline using FlowJo (V-10.2.6).

#### NCAM-1-PEPITEM interactions

Protein Uniprot sequences from the above mass spectrometry were ran alongside PEPITEM (SVTEQGAELSNEER) to predict PEPITEM-potential binding partner interactions in AlphaFold2_multimer using Google colab^48^^,^^49^ to predict both peptide-protein interactions and binding strength. The predicted local distance difference test (pLDDT) was used to select the models with the highest predicted confidence, which were further analyzed using ChimeraX^29^^,^^50^ to create a 3-D rendering of the interaction.

#### NCAM1 and RUNX2 staining

Calvarial osteoblasts seeded onto coverslips in 6 well plates (1 x 10^4^ cells/well) were cultured in osteogenic media for 24h. Cells were fixed in 10% buffered formaldehyde for 15 min at room temperature, followed by three washes in PBS. Samples were permeabilised in 0.1% Triton X-100 (Sigma) for 10 min, followed by 1h 1% BSA (Sigma) blocking at room temperature. Samples were stained with an anti-NCAM1 (AB5032, 2 μg/mL in PBS) and anti-RUNX2 antibody (MAB2006, 10 μg/mL in PBS, Bio-Techne) for 3 h at room temperature, washed three times in PBS before incubation with secondary antibody (1:2000; ThermoFisher Scientific, cat: A11081 and 1:4000; ThermoFisher Scientific, cat: A11034) for 40 min at room temperature in the dark, washed as above and mounted with DAPI mounting media (Invitrogen) and dried overnight prior to storage at −20°C. Images were taken on a ZEISS LSM 780 confocal microscope and analyzed using Zen Blue software (ZEISS).

#### β-catenin signaling

Calvarial osteoblasts seeded onto coverslips in 6 well plates (1 x 10^4^ cells/well) were cultured in osteogenic media for 24 h before treating with PEPITEM (10 ng/mL, for up to 1 h) or lithium chloride (20 mM for 3 h). Cells were fixed in 10% buffered formaldehyde for 15 min at room temperature, followed by three washes in PBS. Samples were stained with a polyclonal β-catenin primary antibody (4 μg/mL in PBS, Merck, cat: 06–734) for 2h at room temperature. Coverslips were washed three times in PBS before incubation with secondary antibody (1:4000; ThermoFisher Scientific, cat: A21447) for 40 min at room temperature in the dark. Coverslips were washed as described before being gently removed from the wells and excess liquid removed. Coverslips were mounted using DAPI mounting media (Invitrogen), dried overnight prior to storage at −20°C. Images were taken on a ZEISS LSM 780 confocal microscope and analyzed using Zen Blue software (ZEISS). Using ImageJ FIJI software (v2.3.1/1.53f), masks were created for individual cells and their nuclei. Relative intensity was measured in both the whole cell and DAPI positive regions, DAPI measurements were then subtracted from the whole cell recordings. Data presented as average integrated density per nucleus or per 100 μm cell area.

#### Osteoclast resorption assays

Murine bone marrow osteoclast precursor cells (10^6^ cells/well) or RAW264.7 cells (8x10^3^cells/well) were seeded onto Osteoassay plates (Corning Inc.) or standard 48-well plates and cultured in either basal media or murine osteoclastogenic media [murine basal media supplemented with 50 or 10 ng/mL RANKL (Abcam, cat; Ab129136)] for up to 7 days. Alternatively, primary human monocytes were seeded into 12-well (10^6^ cells/well) or 96-well plates (2 x 10^5^ cells/well) containing small dentine disks in human osteoclastogenic media (human osteoclast basal media supplemented with 25 ng/mL *m*-CSF and 25 ng/mL RANKL (both R&D systems). In some cases, media was supplemented with either: 10 ng/mL PEPITEM, 10 ng/mL control peptide, or 1 μM zoledronic acid hydrate (Cambridge Biosciences).

Osteoclast differentiation was assessed by measuring TRAP using a method similar to that described above. Briefly, cultured cells were fixed in 10% PFA for 15 min and then incubated with the TRAP staining solution for 30 min at 37^o^C. Wells were washed three times in distilled water before imaging using the Cytation5 microscope (Agilent) with BioTek Gen5 software. Number of osteoclasts per well were analyzed using ImageJ and expressed as osteoclasts per 100 m^2^.

Osteoclasts seeded into OsteoAssay plates were removed by a 5 min incubation in 10% H_2_O_2_. Plates were washed in PBS, allowed to air dry and imaged using the Cytation5 microscope with Gen5. Hydroxyapatite resorption was then analyzed in ImageJ FIJI by color thresholding areas of resorption and calculating the percentage of resorption per area/well.

Dentine disks were treated with 1M NH_4_OH for 24h, washed and air dried. Dentine resorption pits were stained with 0.5% Toluidine Blue in 0.5% Boric acid for 2min, before being washed, air dried and mounted onto slides using DPX mountant (Sigma-Aldrich). z stack images of the dentine were acquired using the Axioscan Z1 slide scanner (ZEISS). A manual mask of the stained resorption pits was created using ImageJ FIJI software allowing for resorption area to be measured. Data were expressed as percentage of total dentine disk resorbed.

#### Secretome assays

RAW264.7 (6-8 x 10^3^ cells/well) were cultured with 50 ng/mL RANKL in basal murine osteoblast media or conditioned media from treated osteoblasts for 5 days, prior to staining for TRAP (as above). Prior to addition to RAW264.7 cells, conditioned media from PEPITEM-treated osteoblasts was supplemented with 50 ng/mL RANKL along with either: 0.15 or 0.5 μg/mL OPG polyclonal neutralising antibody (cat: AF459, Biotechne, UK) or an IgG control antibody (0.5 μg/mL, Invitrogen, cat: 02–6102). In competition assays, conditioned media was supplemented with 50, 100 or 500 ng/mL RANKL (Abcam).

#### ELISA

Osteoprotegerin (OPG) or RANKL concentrations (pg/mL) in osteoblast supernatants were measured in technical duplicates using R&D Quantikine ELISA kits as per manufacturer’s instructions (R&D Systems) using a Synergy HT plate reader with absorbance set at 405nm. Data were expressed as a percentage of the untreated control. Serum was analyzed for RatLaps total procollagen type-1 N terminal propeptide (P1NP) by a competitive ELISA as per the manufacturer’s instructions and read using a Synergy HT plate reader at 405nm. A 4- parameter logistical curve fit was applied to the data to calculate P1NP serum concentrations (ng/mL).

#### Gene expression

RNA was extracted using the RNeasy Kit as per manufacturer’s instructions (Qiagen). 500ng RNA was converted to cDNA using a high-capacity cDNA reverse transcription kit following the manufacturer’s instructions (Applied Biosystems). Gene-specific Applied Biosystems Assay-on-Demand TaqMan primers were diluted 1:10 in master mix (Applied Biosystems), before plating into 384 LightCycler plates along with 2.75 μL of diluted cDNA (1:5) in diH2O. Samples were run in technical triplicates and analyzed on the LightCycler 480. Data were normalised to β_2_-microglobin and expressed as 2^−ΔCt^.

Alternatively, library preparation was performed on samples with RIN of >7 using the Lexogen QuantSeq 3′ mRNA-Seq Library Prep Kit FWD for Illumina (Illumina, California). Samples that passed library prep were pooled in equal volumes and quality checked by the Genomics service using the Agilent TapeStation DNA 1000 tape (Aligent) and DNA HS Qubit (Fisher Scientific). Sequencing was then performed using the NextSeq 500 (Illumina), where the run had a Yield (Gbp) of 43.58 and an average %Q30 of 91.17, indicating a successful run and analysis could be performed in house.^51^^,^^52^ Data generated were provided in fastq format, where quality of each sample could be checked using FASTQC and MULTIQC packages in Rstudio (RStudio Public-benefit corporation).^53^ Four reads were generated for each sample, which were combined and subsequently trimmed. Trimming was performed using BBMap to remove the polyA tail, low quality RNA (Using the Phred quality score algorithm) and contaminating adaptor regions.^54^

In order to align the genome, a genome index was created using a pre-assembled genome from ensembl.^55^ The “Mus_musculus.GRCm39.104.gtf” genome and corresponding primary assembly file were used to create the genome used for our analysis. Trimmed sequences were then aligned to the genome using the STAR align package and the Encode standard settings.^56^ MultiQC was performed on FastQC files generated from the aligned samples and each parameter was checked to ensure quality of pre-processing. Feature counts were produced using the Subread package to generate a table of gene counts.^57^^,^^58^ The produced feature counts were then loaded and analyzed using iDep.^59^ IDep correctly identified the samples as *Mus musculus* and rlog transformation was selected. Genes were then ranked by standard deviation using hierarchical clustering. Samples were then plotted on a PCA plot using first and second principal components. The Dseq2 package was then used to identify upregulated and downregulated genes. Genes were analyzed using GO term analysis.

### Quantification and statistical analysis

Data are expressed as mean ± SEM unless otherwise stated. To ensure the data met the assumptions for each statistical test, normality was assessed using Kolmogorov-Smirnov test. Univariant analysis was performed using unpaired t-test, paired t-test or Wilcoxon signed-rank test. Multi-variant analysis was performed using ANOVA or Kruskal-Wallis followed by Dunnett’s, Bonferroni or Dunn post-hoc test. For the purposes of visualisation (∗) *p* < 0.05 was deemed to be statistically significant. The statistical parameters, including the value of n and what n entails, are reported in the relevant methods sections and within each figure legend.

## Supplemental Information

Document S1. Figures S1–S7 and Tables S1–S5

Figure360. An author presentation of Figure 6

Document S2. Article plus supplemental information
