# Therapeutic avenues in bone repair: Harnessing an anabolic osteopeptide, PEPITEM, to boost bone growth and prevent bone loss

**DOI:** 10.1016/j.xcrm.2024.101657

**Published:** 2024-07-09

**Authors:** Jonathan W. Lewis, Kathryn Frost, Georgiana Neag, Mussarat Wahid, Melissa Finlay, Ellie H. Northall, Oladimeji Abudu, Samuel Kemble, Edward T. Davis, Emily Powell, Charlotte Palmer, Jinsen Lu, G. Ed Rainger, Asif J. Iqbal, Myriam Chimen, Ansar Mahmood, Simon W. Jones, James R. Edwards, Amy J. Naylor, Helen M. McGettrick

## Main text

(Cell Reports Medicine *5*, 101574; May 21, 2024)

As a result of an oversight in the originally published version of this article, Samuel Kemble was not included in the author list. This error has now been corrected in the article online. The authors regret this error.

