# Supplementary material for: Therapeutic avenues in bone repair: Harnessing an anabolic osteopeptide, PEPITEM, to boost bone growth and prevent bone loss
Source: Cell Rep Med. 2024 May 21;5(5):101574. doi: 10.1016/j.xcrm.2024.101574 (PMC11148860; doi:10.1016/j.xcrm.2024.101574)
Supplement: Document S1. Figures S1–S7 and Tables S1–S5 [file mmc1.pdf]

Cell Reports Medicine, Volume *5*

Supplemental information

Therapeutic avenues in bone repair: Harnessing an anabolic osteopeptide, PEPITEM, to boost bone growth and prevent bone loss

Jonathan W. Lewis, Kathryn Frost, Georgiana Neag, Mussarat Wahid, Melissa Finlay, Ellie H. Northall, Oladimeji Abudu, Samuel Kemble, Edward T. Davis, Emily Powell, Charlotte Palmer, Jinsen Lu, G. Ed Rainger, Asif J. Iqbal, Myriam Chimen, Ansar Mahmood, Simon W. Jones, James R. Edwards, Amy J. Naylor, and Helen M. McGettrick

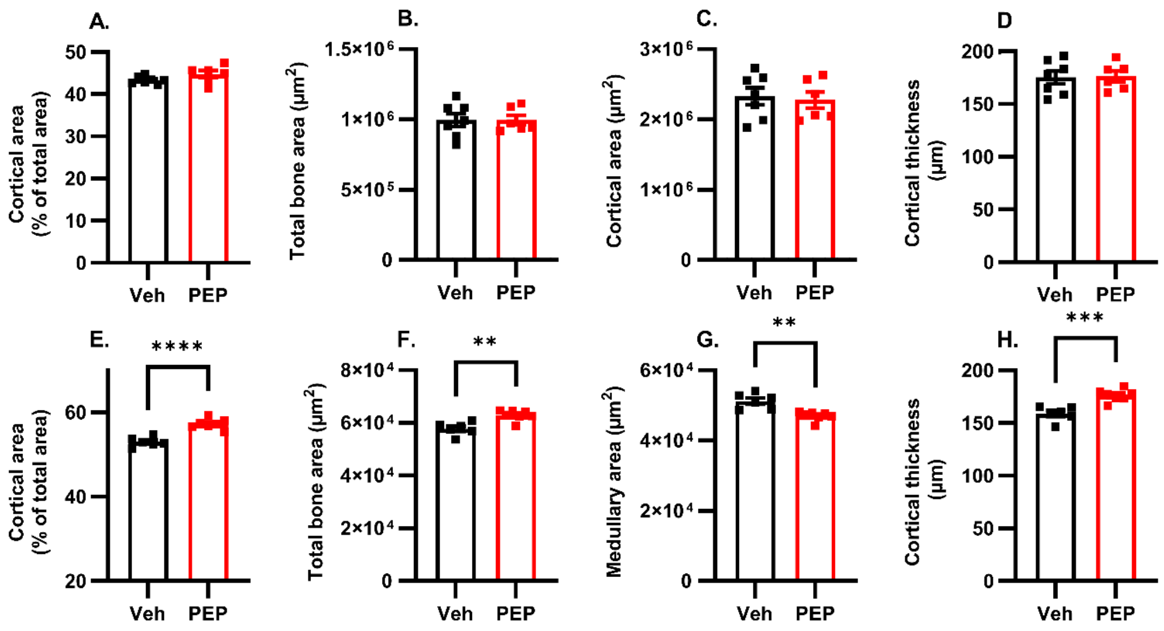

Figure S1: PEPITEM increased cortical bone parameters under homeostatic conditions, related to Figure 1: Epiphyseal cortical bone from the tibiae from healthy young mice injected with vehicle control (Veh, black, n=6-7) or PEPITEM-PEG (PEP, red, n=6) daily for **(A-D)** 14 or **(E-H)** 28 days were analysed by microCT. **(A,E)** Percentage of cortical area. **(B,F)** Total bone area in μm^2. **(C,G)** Total cortical area in μm^2. **(D,H)** Cortical thickness in µm. Data are mean ± SEM for 3 independent experiments. **=p<0.01, ***=p<0.001 and ****=p<0.0001 by unpaired t-test.

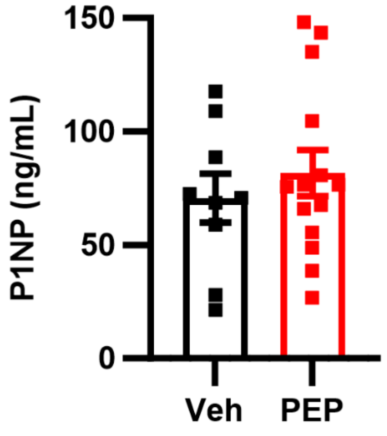

Figure S2: PEPITEM has no effect on serum levels of P1NP, related to Figure 1. Healthy young mice injected with vehicle control (Veh, black, n=9) or PEPITEM-PEG (PEP, red, n=14) and serum levels of P1NP were analysed by ELISA and expressed as ng/ml. Data are mean ± SEM.

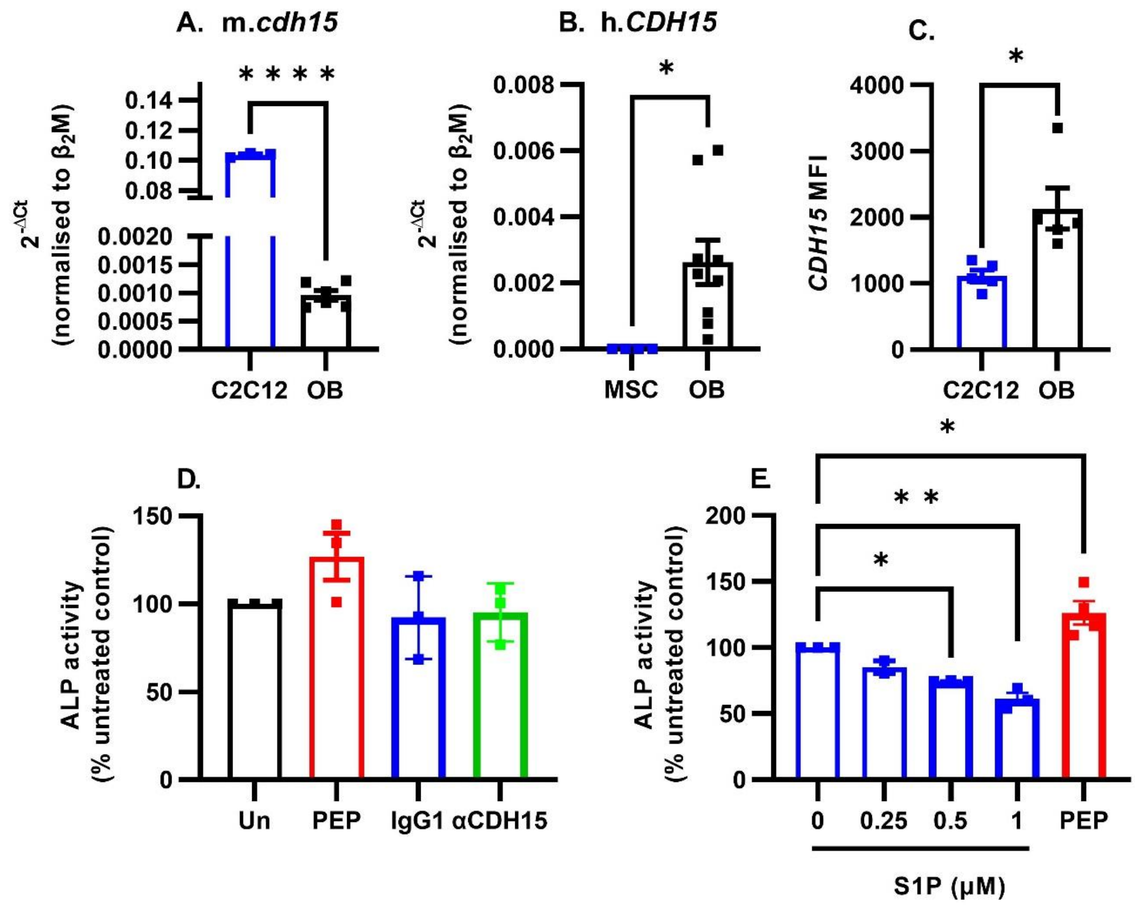

Figure S3: PEPITEM signalling in osteoblasts is not mediated by cadherin-15 or sphingosine-1-phosphate, related to Figure 3.

Gene and protein expression of cadherin 15 in **(A,C)** murine muscle cell line (C2C12, blue, n=3-5), primary murine calvarial osteoblasts (OB, black, n=5-6) or **(B)** primary mesenchymal stem cells (MSC, blue, n=4) or human synovial osteoblasts (OB, black, n=9). (A-B) Cadherin-15 gene expression normalised to beta-2-microglobin and expressed as $2^{-\Delta Ct}$. Cadherin-15 protein expression as (C) median fluorescence intensity (MFI). **(D)** Murine osteoblasts (n=3) were cultured in osteogenic media along (untreated, Un, black) or supplemented with PEPITEM (PEP, red), or IgG1 control (IgG1, blue), or agonist antibody against cdh15 (αCDH15, green). **(E)** Murine osteoblasts (n=3) were cultured in increasing concentrations of exogenous sphingosine-1-phosphate (S1P, black) or supplemented with PEPITEM (PEP, red). Data are mean ± SEM. * = p< 0.05, and **=p<0.01 by Dunnett post-test.

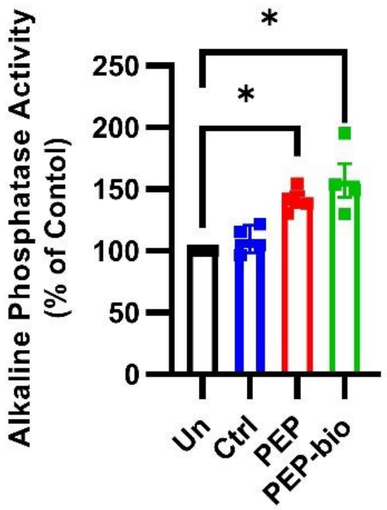

Figure S4: Identifying PEPITEM binding partners, related to Figure 4. Primary calvarial osteoblasts were cultured in osteogenic media (untreated, Un, black) supplemented with PEPITEM (PEP, red) or biotinylated PEPITEM (PEP, blue). Alkaline phosphatase activity for osteoblasts measured at day 8 and normalised to percentage of untreated control (n=4). Kruskal-Wallis shows a significant effect of treatment, $p<0.001$. Data are mean ± SEM. * = $p< 0.05$ by Dunn post-test.

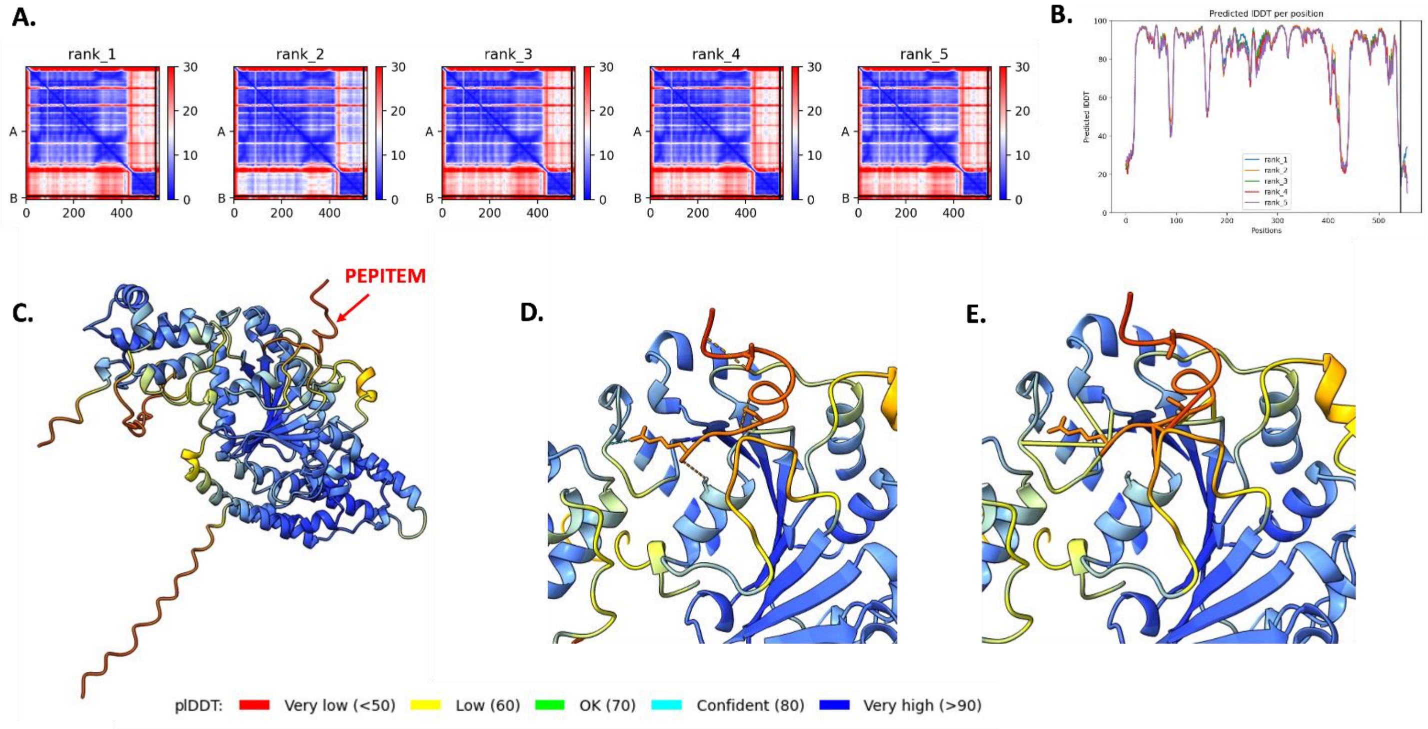

Figure S5: 3-D predictive modelling of PEPITEM interaction with EHD2, related to Figure 4. Full length EHD2 sequence was ran with PEPITEM in (A) AlphaFold-Multimer followed by analysed of rank 1 model (B-D) using ChimeraX. **(A)** Predicted aligned error heat map for five models of EHD2 and PEPITEM, where blue and red indicate low or high error, respectively. **(B)** Graph representing predicted pLDDT of each residue in the 5 models, ranked 1-5. **(C)** ChimeraX modelling of rank 1 model for PEPITEM interactions with EHD2 coloured by pLDDT score of 70>50. **(D-E)** Magnified view of binding location of PEPITEM on EHD2 revealing **(D)** 3 predicted hydrogen bonds and **(E)** 12 pseudobonds - high likelihood (blue) and low likelihood (red), confidence indicated by pLDDT colour.

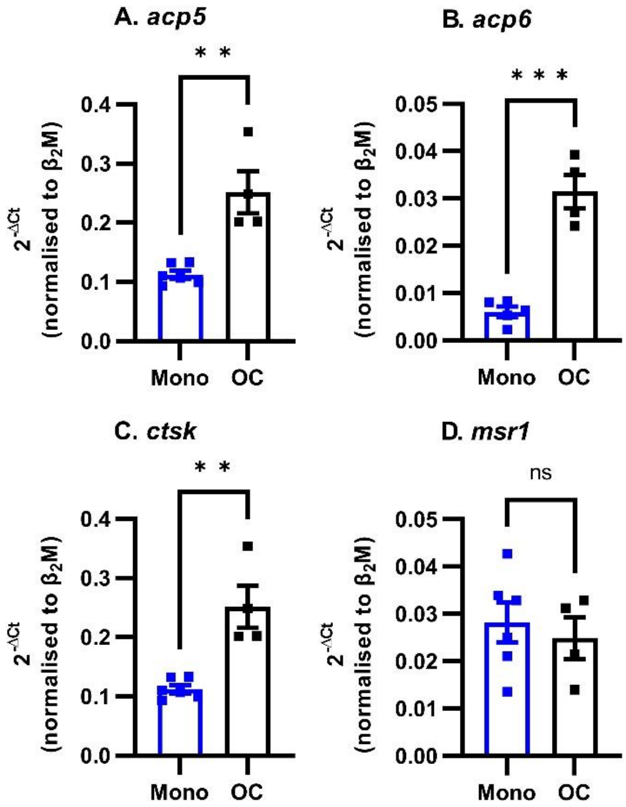

Figure S6: RANKL-induced osteoclast gene expression in human primary osteoclasts, related to Figure 5. Human peripheral blood monocytes treated without (mono, blue, n=6) or with RANKL and M-CSF (OC, black, n=4). Gene expression for the osteoclast specific genes **(A)** *ACP5,* **(B)** *ATP6V1B1* **(C)** *CTSK* and the monocyte specific gene **(D)** *MSR1* were normalised to beta-2-microglobin and expressed as $2^{-\Delta Ct}$. Data are mean ± SEM. **=p<0.01 and ***=p<0.001 by unpaired t-test.

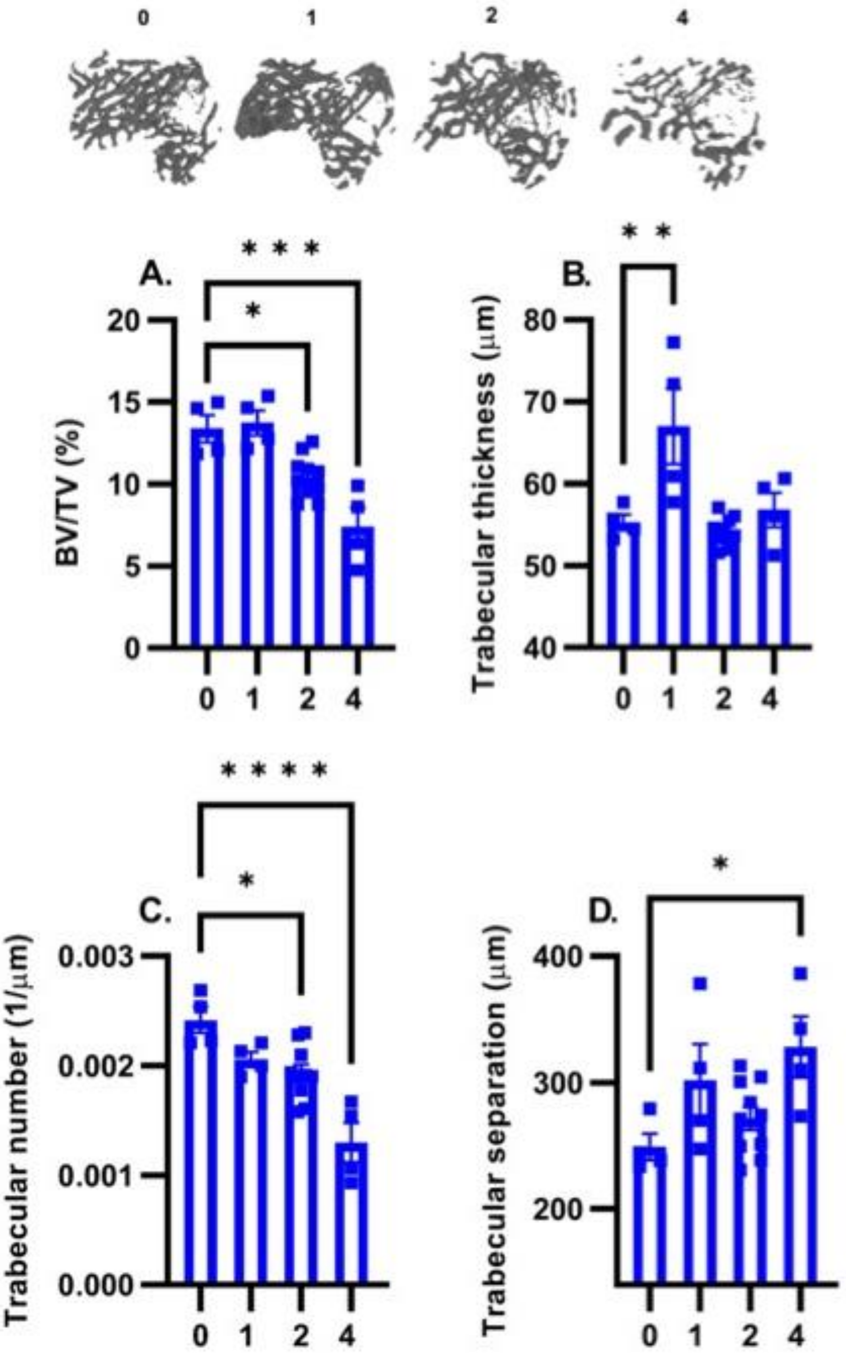

Figure S7: Ovariectomy induces bone loss over 4 weeks, related to Figure 7. Ovariectomy was performed and bones collected at baseline (0, n=4), 1-week (n=4), 2-weeks (n=10) and 4-weeks (n=4) post-OVX. Representative 3D microCT renders of trabecular bone from tibiae taken at each time point. **(A)** Percentage trabecular bone volume (BV/TV). **(B)** Trabecular thickness in µm. **(C)** Trabecular number per µm. **(D)** Trabecular separation in µm. ANOVA showed significant effect of time on all bone parameters, $p<0.05$. Data are mean ± SEM from 3 independent experiments. * = $p<0.05$, ** = $p<0.01$, ***=$p<0.001$ and ****=$p<0.0001$ by Dunnett's post-test compared to baseline time zero samples.

Table S1: Top 10 hits from PEPITEM-co-IP, related to Figure 3.

Rank	Description	Gene ID	Ave. Peptides	MW (kDa)	calc. pI	Ave. Sequest HT Score
1	Annexin A6	*ANXA6*	25.5	75.8	5.5	110.9625
**2**	**Neural cell adhesion molecule 1**	***Ncam1***	**14.75**	**93.6**	**4.93**	**61.91**
3	Protein transport protein Sec31A	*Sec31a*	17	133.5	6.76	49.5933
4	EH domain-containing protein 2	*Ehd2*	16	61.1	6.51	48.0233
5	Catenin delta-1	*Ctnnd1*	16	108.1	6.23	46.8633
6	Catenin alpha-1	*Ctnna1*	11.67	100	6.23	38.6767
7	Dipeptidyl peptidase 3	*Dpp3*	10.33	82.8	5.34	33.0067
8	Serine/threonine-protein phosphatase 2A 65 kDa regulatory subunit A beta isoform	*Ppp2r1b*	10.33	76	5.19	26.8033
9	Pleckstrin homology domain containing, family C (with FERM domain) member 1	*Fermt2*	11.5	77.8	6.7	23.7
10	Nucleoredoxin	*Nxn*	10.33	48.3	4.93	20.2133

calc. pI = calculated isoelectric point

Table S2: Differentially expressed genes up-regulated in PEPITEM treated osteoblasts at 6h compared to osteoblasts treated with control peptide, related to Figure 3.

Symbol	Chr	log2 Fold Change
Kmt2d	15q	1.40430776556747
Flt1	5q	1.15024565487949
Cpeb2	5q	1.05760092791329
Rbm45	2q	1.0073207049212
Lama2	**10q**	**0.996559007758521**
Ptprm	17q	0.952178517843655
Hivep2	10q	0.947823623371367
Eng	2q	0.934226403521769
Wdfy3	5q	0.901213704744887
Fem1c	18q	0.89702569683231
Dip2b	15q	0.881391238649078
Zbtb20	16q	0.877883505427051
Dram1	10q	0.866023036178446
Patl1	19q	0.862128858338649
Zfp568	7q	0.846745380410452
Trrap	5q	0.83225947069035
Arl13b	16q	0.821490969991678
Rassf3	10q	0.818505822258442
Zfp462	4q	0.798118146421969
Brca2	5q	0.791758570825744
Pcnx3	**19q**	**0.78673474216748**
Arid1a	4q	0.752321141728179
Nf1	**11q**	**0.728727334748264**
Sacs	14q	0.728490160061874
Nav2	7q	0.71317444560951
Ubr4	**4q**	**0.706757934426878**
Zfhx4	**3q**	**0.698144342411609**
Usp24	4q	0.684570755941333
Dst	1q	0.681690402221993
Nav1	1q	0.680660086659274
Diaph2	Xq	0.676263269282805
Thsd7a	6q	0.671998492935553
Ahnak	19q	0.664900807472944
Sp1	**15q**	**0.656539085200315**
Xrn1	**9q**	**0.649850897735021**
Heatr5a	12q	0.633982450946088
Phc3	3q	0.624441130291069
Trio	15q	0.615951905744821
Kmt2a	9q	0.606230239442185
Ago3	**4q**	**0.600361833824849**
Pcdh19	Xq	0.600219331861358
Col12a1	**9q**	**0.599971791753937**
Flnc	6q	0.59773839281501
Hipk1	3q	0.58553236973944
Golgb1	16q	0.580697629173566

Dync1h1	12q	0.577300702390066
Lrp1	**10q**	**0.57640041285326**
Birc6	17q	0.572912603471088
Tmem245	4q	0.572343054102517
Prrc2a	17q	0.54450647950633
Phip	9q	0.535110268581313
Uggt1	1q	0.528959887530308
Lpp	16q	0.525533520965761
Fat1	**8q**	**0.518896007730861**
Zfhx3	8q	0.518644419881584
Mki67	7q	0.517627805029924
Ylpm1	12q	0.513172915908124
Dyrk2	**10q**	**0.510496882653584**
Herc2	**7q**	**0.493448659228275**
Macf1	4q	0.489793110321781
Nfib	**4q**	**0.486301541109151**
Sh3pxd2a	19q	0.467458423558138
Pappa	**4q**	**0.457252945009879**
Huwe1	**Xq**	**0.455546160489084**
Purb	11q	0.452468062965651
Golga4	9q	0.439834219941734
Prrc2c	1q	0.416493712928052
Col6a3	1q	0.40514247754676
Ubr5	15q	0.403062904310555
Igf2r	17q	0.397811552206264
Myadm	7q	0.38156757024043
Taok1	11q	0.352772156911008
Nipbl	15q	0.328218940767693
Tead1	7q	0.315132470594774
Col1a1	**11q**	**0.247360900416806**

Genes highlighted in bold have known function in osteoblast differentiation or skeletal development

Table S3: Differentially expressed genes down-regulated PEPITEM treated osteoblasts at 6h compared to osteoblasts treated with control peptide, related to Figure 3.

Symbol	Chr	log2 Fold Change
Rex1bd	8q	-1.23481171180353
Pop5	5q	-1.23010585840839
Mrpl11	19q	-1.17099433291063
Ciao2b	8q	-1.08294378277525
Cir1	2q	-0.998050090509771
Cwc27	13q	-0.987369075921076
Lamtor3	3q	-0.973491012577354
Nab2	10q	-0.970298295696761
Ccnt2	1q	-0.944754965505827
Dhrs7b	11q	-0.902132781899503
Ubp1	9q	-0.822108448415377
Rnf7	9q	-0.822100913458168
Dhx40	11q	-0.802539677742481
Gm8292	1q	-0.79510122822112
Cenpw	10q	-0.789939896231761
Nudt16l1	16q	-0.788201129897281
Dhx30	9q	-0.769932193365714
Lamtor5	3q	-0.733875307178892
Med10	13q	-0.72561370033205
Snhg18	15q	-0.684307667385217
Tk1	11q	-0.62883995396853
Ndufb11	Xq	-0.595363528291204
Mrpl52	14q	-0.514535959989639
Tnmd	Xq	-0.489570358081693
Rps11	7q	-0.481282814956995
Pcmt1	10q	-0.480143879032629
Lrrc59	11q	-0.479872476259173
Crk	11q	-0.455460664332768
Rps23	13q	-0.441999041257278
Nol7	13q	-0.439943599153777
Gpx1	9q	-0.430077387508551
Rps19	7q	-0.421563308898329
Oxct1	15q	-0.418087519788854
Atp6v0e	17q	-0.412833683151441
Atpif1	4q	-0.394691300295738
Rpl22	4q	-0.354813108044449

Table S4: AlphaFold-Multimer predicted binding residues for PEPITEM interacting with NCAM-1, related to Figure 4.

**Binding Protein**	Bond Type	Protein residue	PEPITEM residue	Distance (Å)	pLDDT range score
**NCAM1**	Hydrogen Bond	LYS 619 NZ	SER 10 OG	3.234	N/A
		SER 656 OG	GLY 6 O	2.746	N/A
		HIS 658 ND1	SER 10 N	3.017	N/A
		LEU 622 O	ASN 11 ND2	2.837	N/A
	Predicted Pseudobonds	SER 656	LEU 9	3	50-70
		SER 656	GLLY 6	3	50-70
		SER 656	GLU 8	3	50-70
		SER 656	ALA 7	3	50-70
		PRO 653	GLY 6	3	50-70
		PRO 653	ALA 7	3	50-70
		ASP 657	ASN 11	3	50-70
		ASP 657	SER 10	3	50-70
		ASP 657	GLU 8	3	50-70
		ASP 657	LEU 9	3	50-70
		HIS 658	SER 10	3	50-70
		HIS 658	LEU 9	3	50-70
		HIS 658	GLU 8	3	50-70
		LEU 622	ASN 11	3	50-70
		LEU 622	LEU 9	3	50-70
		GLY 655	ALA 7	3	50-70
		GLY 655	GLU 8	3	50-70
**Fibronectin-III Domains (from NCAM1)**	Hydrogen Bond	VAL 37 N	LEU 9 O	2.676	N/A
		ILE 39 N	ALA 7 O	2.755	N/A
		THR 71 OG1	GLN 5 OE1	3.358	N/A
		ASP 102 N	VAL 2 O	2.818	N/A
		ASP 102 OD1	THR 3 OG1	3.125	N/A
		ASP 102 OD2	THR 3 OG1	3.170	N/A
		ASP 101 OD1	GLU 4 N	2.499	N/A
		VAL 37 O	LEU 9 N	2.583	N/A
		GLY 35 O	ASN 11 N	3.341	N/A
	Predicted Pseudobonds	ASP 102	SER 1	3	70-90
		ASP 102	VAL 2	3	70-90
		ASP 101	VAL 2	3	70-90
		ASP 101	GLU 4	3	70-90
		TYR 9	ASN 11	3	70-90
		MET 40	VAL 2	3	>90
		PRO44	GLN 5	3	>90
		LYS 43	GLN 5	3	>90
		ILE 39	GLY 6	3	>90
		ILE 39	ALA 7	3	>90
		ILE 39	LEU 9	3	>90
		VAL 50	LEU 9	3	>90
		VAL 37	LEU 9	3	>90

pLDDT score = predicted local distance difference test score

Table S5: Demographic information for aged patients, related to STAR methods section - Experimental model and study participant details.

	**Cohort (n=10)**
**Age (years) †**	67 (59.25-77.50)
**Female; number (%)**	90 (9)
**Clinical Diagnosis**	Osteoarthritis

†median (interquartile range)
